# Macrophagic CD146 promotes foam cell formation and retention during atherosclerosis

**DOI:** 10.1038/cr.2017.8

**Published:** 2017-01-13

**Authors:** Yongting Luo, Hongxia Duan, Yining Qian, Liqun Feng, Zhenzhen Wu, Fei Wang, Jing Feng, Dongling Yang, Zhihai Qin, Xiyun Yan

**Affiliations:** 1 Key Laboratory of Protein and Peptide Pharmaceutical, Institute of Biophysics, Chinese Academy of Sciences, Beijing 100101, China; 2 Beijing Anzhen Hospital of the Capital University of Medical Sciences, Beijing 100029, China

**Keywords:** foam cell formation, CD146, CD36, retention, atherosclerosis

## Abstract

The persistence of cholesterol-engorged macrophages (foam cells) in the artery wall fuels the development of atherosclerosis. However, the mechanism that regulates the formation of macrophage foam cells and impedes their emigration out of inflamed plaques is still elusive. Here, we report that adhesion receptor CD146 controls the formation of macrophage foam cells and their retention within the plaque during atherosclerosis exacerbation. CD146 is expressed on the macrophages in human and mouse atheroma and can be upregulated by oxidized low-density lipoprotein (oxLDL). CD146 triggers macrophage activation by driving the internalization of scavenger receptor CD36 during lipid uptake. In response to oxLDL, macrophages show reduced migratory capacity toward chemokines CCL19 and CCL21; this capacity can be restored by blocking CD146. Genetic deletion of macrophagic CD146 or targeting of CD146 with an antibody result in much less complex plaques in high-fat diet-fed ApoE^−/−^ mice by causing lipid-loaded macrophages to leave plaques. Collectively, our findings identify CD146 as a novel retention signal that traps macrophages within the artery wall, and a promising therapeutic target in atherosclerosis treatment.

## Introduction

Atherosclerosis has been considered as a non-resolving autoinflammatory disease of the arterial walls^1,2^. As the main contributor of cardiovascular events, such as myocardial infarction and stroke, atherosclerosis is one of the leading causes of death worldwide^3^. The typical pathology of atherosclerosis is the formation of atheroma containing a large number of necrotic cells and inflammatory cells. Macrophages, especially ones that engorge cholesterol (foam cells) cause the formation of atheroma^4^. It has been reported that the compound retention of foam cells in the subintima is a fundamental step of plaque initiation and development^5,6^. The retarded foam cells facilitate the adventitia angiogenesis and the build-up of necrotic pools, resulting in atherosclerosis exacerbation and plaque instability. Unlike other resolving inflammatory conditions, inflammation in atherosclerotic plaque does not easily resolve, largely due to the abundant cholesterol-engorged macrophages that became immotile and trapped in the plaque under hyperlipidemia^7,8^.

Although the accumulation of macrophages in the artery wall has long been considered a major inducer of chronic inflammation, the mechanism that regulates formation of macrophage foam cells and their retention within the atheroma remains largely unknown^4,7^. Macrophages can emigrate at the early plaque stage^9^ or during atherosclerosis regression, but they become foam cells and gradually lose the capacity for emigration during progression of the atherosclerotic lesion^6,7,10^. There is growing evidence supporting the notion that the balance between retention and emigration signals contributes to the accumulation of macrophages in plaques. Researchers are only beginning to elucidate the regulatory signals that control macrophage emigration or retention^6,7^. Studies have shown that the uptake of oxidized low-density lipoprotein (oxLDL) mediated by scavenger receptor CD36 accounts for a large proportion of the formation of macrophage foam cells and their retention in atherosclerotic lesions^11,12^. Blocking the oxLDL uptake or normalizing high-density lipoprotein (HDL) decreases the number of CD68-positive macrophages in the plaques^13,14^. Moreover, activation of the chemokine receptor CCR7-dependent emigration pathway in macrophages promotes regression of atherosclerosis^15,16^. Recent studies revealed that the upregulation of neuroimmune guidance cues, netrin-1 or semaphorin 3E, under hypoxic conditions exacerbates plaque inflammation by promoting macrophage retention^17,18,19^. Therefore, inhibiting the formation of foam cells or restoring their capacity for emigration should facilitate resolution of chronic inflammation and improve clinical outcomes among patients with atherosclerosis^4,6^. Thus, identification of the underlying mechanisms that control foam cell formation and retention within the plaque may lead to the discovery of novel therapeutic targets in atherosclerosis^2^.

Pattern recognition receptor CD36 is an essential component of foam cell formation and atherosclerosis^7^. In ApoE^−/−^ mice, the absence of CD36 protects them from the development of atherosclerosis and lesion complexity^20,21,22^. CD36 functions as a principal receptor responsible for the uptake of oxLDL, thereby leading to lipid loading and foam cell formation. The internalization of CD36 initiated by stimulation with oxLDL not only is important for lipid uptake but also triggers signaling cascades for inflammatory responses. For efficient internalization, oxLDL and CD36 require the recruitment of membrane receptors, adaptor proteins and the cytoskeleton to form heteromultimeric complexes^23^. Functionally, oxLDL via CD36 contributes to the loss of cell polarity and decreases migratory capacity of macrophages; these changes may represent a macrophage-trapping mechanism in the development of atherosclerosis^12,24^. Although the uptake of oxLDL by CD36 has been confirmed to be a critical step for foam cell formation, the mechanism regulating ligand-induced CD36 internalization and its corresponding downstream signals remain poorly defined. Clarification of this regulatory mechanism may lead to a new treatment strategy for atherosclerosis^23^.

CD146 (also known as MCAM) is a member of the immunoglobulin superfamily that was originally identified as a melanoma marker^25^. Several reports showed that CD146 is associated with the development of many non-resolving inflammatory diseases, including rheumatoid arthritis, multiple sclerosis, asthma and inflammatory bowel disease^26,27,28^. CD146 is detected on activated inflammatory cells, including a subset of lymphocytes and alveolar macrophages, indicating that CD146 expression might confer a pro-inflammatory property on immune cells^29,30^. We have previously reported that CD146 is expressed on infiltrated macrophages of human atherosclerotic plaques, and is associated with the plaque vulnerability^31^, indicating that CD146 expression on plaque macrophages might have a role in the atherosclerosis progression. Given its function in immune cell activation and inflammatory diseases, we sought to determine its role on atherosclerosis development, especially the role on formation of foam cells and their retention.

In the present study, we found that CD146 is expressed on macrophage foam cells in human and mouse atheroma. We demonstrated that CD146 is upregulated by oxLDL and promotes foam cell formation by mediating CD36 internalization during lipid uptake, thereby inhibiting macrophage motility. Deletion of macrophagic CD146 or targeting of CD146 with specific antibody inhibits atherosclerosis development through reducing foam cell formation and restoring the emigration processes of oxLDL-loaded macrophages. Our findings suggest that macrophagic CD146 has an active role in the pathogenesis of atherosclerosis and may be a novel therapeutic target for atherosclerosis treatment.

## Results

### Macrophage-derived foam cells express CD146

**Figure 1:**
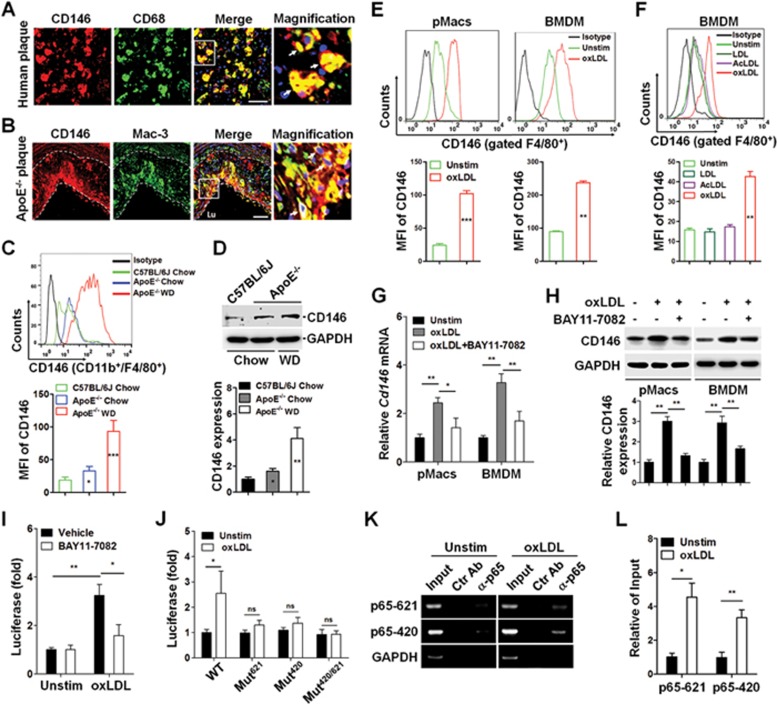
CD146 is upregulated in macrophage foam cells. **(A)** Human carotid artery (*n* = 3) atherosclerotic lesions staining for CD146 (red) and CD68 (green) and their co-localization (yellow merge; see arrows). **(B)** Atherosclerotic plaques from ApoE^−/−^ mouse (*n* = 5) that was fed a Western diet (WD) for 18 weeks staining for CD146 (red) and Mac-3 (green) and their co-localization (yellow merge; see arrows). The nuclei were stained with DAPI (blue). The dashed lines indicate the lesion borders. The scale bars in **A** and **B** are 50 μm. **(C**, **D)** Flow cytometric analysis **(C)** or western blot **(D)** of CD146 expression in CD11b^+^F4/80^+^ peritoneal macrophages isolated from wild-type C57BL/6J mice fed a normal diet (chow) or ApoE^−/−^ mice (*n* = 5) fed a normal diet or a WD. Bottom, quantification of the mean fluorescent intensity (MFI) of CD146 in each group (*n* = 5). CD146 expression in **D** (bottom) is presented relative to that of GAPDH (loading control). **(E)** Flow cytometric analysis of CD146 expression in F4/80^+^ peritoneal macrophages and bone marrow-derived macrophages (BMDMs) that were treated with or without oxLDL (50 μg/ml) for 24 h. Bottom panel: quantification of the MFI of CD146 in each group (*n* = 5). **(F)** Flow cytometric analysis of CD146 expression in F4/80^+^ BMDMs that were treated with LDL, acetylation LDL (AcLDL) or oxLDL (50 μg/ml) for 24 h. Bottom panel: quantification of the MFI of CD146 in each group (*n* = 5). **(G**, **H)** Real-time PCR analysis of mRNA level of *Cd146* **(G)** or western blot analysis of CD146 protein level **(H)** in peritoneal macrophages and BMDMs that were treated with oxLDL (50 μg/ml) for 24 h in the presence or absence of NF-κB inhibitor BAY11-7082 (20 μM) (*n* = 3). **(I)** *Cd146* promoter-luciferase reporter activity in HEK293 cells treated with oxLDL (50 μg/ml) in the presence or absence of the NF-κB inhibitor, presented relative to luciferase activity in unstimulated cells, set as 1. **(J)** Dual luciferase assay of putative NF-κB binding sites in the *Cd146* promoter. The luciferase activity of these constructs was measured and normalized to that of the unstimulated wild-type construct (pGL3-WT) (*n* = 5). **(K)** ChIP assay of p65 binding to *Cd146* promoter using an anti-p65 antibody or an isotypic control, followed by PCR amplification of the genomic DNA fragments covering the binding site -621 and -420. GAPDH was used as the internal control. **(L)** Quantification of the relative level of PCR product to that of input. Two-way ANOVA test, ^*^P < 0.05, ^**^P < 0.01, ^***^P < 0.001. The data represent three independent experiments.

To investigate whether macrophagic CD146 is involved in the development of atherosclerosis, we first tested its expression on the macrophage foam cells in atherosclerotic plaques of carotid artery from both human and Western diet-fed ApoE^−/−^ mice, an animal model for atherosclerosis. Human macrophages were stained with antibodies against CD146 and CD68 (a marker for human macrophages). Expression of CD146 was observed on macrophage foam cells labeled by CD68 in atherosclerotic plaques from patients (Figure 1A). Consistent with this result, the ApoE^−/−^ mice that had been fed a Western diet for 18 weeks, displayed a similar staining pattern of CD146 and Mac-3 (a marker for murine macrophages) in their aortic plaques (Figure 1B). These data indicate that the expression of CD146 on macrophage foam cells is a common feature of mouse and human atheroma.

To figure out whether the expression of CD146 on macrophages is regulated by lipoproteins, we first isolated peritoneal macrophages (F4/80^+^CD11b^+^) from wild-type (WT) C57BL/6J mice and ApoE^−/−^ mice that were fed either regular chow or a Western diet; the latter is a well-established model of *in vivo* foam cell formation. As detected by flow cytometry (Figure 1C) and immunoblot (Figure 1D), CD146 was expressed at a higher level on the macrophages from the Western-diet-ApoE^−/−^ mice than that from the chow diet-ApoE^−/−^ mice and normal mice, which implied that the expression of CD146 might be related to lipid accumulation in macrophages. To further assess this observation, we used a principal component of cholesterol, including LDL, oxLDL or acetylated LDL (AcLDL) to treat peritoneal macrophages or bone marrow-derived macrophages (BMDMs) from C57BL/6J mice, and examined CD146 expression using flow cytometry. We found that only oxLDL, but not LDL or AcLDL, upregulated CD146 expression on both peritoneal macrophages (Figure 1E) and BMDMs (Figure 1F). These data suggest that the expression of CD146 on macrophage could be regulated by oxLDL, an important contributor to atherosclerosis.

The binding of oxLDL to its receptor CD36 activates transcription factor NF-κB, whereas the *Cd146* promoter contains two NF-κB-binding sites (Supplementary information, Figure S1); we therefore tested whether NF-κB contributes to oxLDL-induced CD146 upregulation. Notably, the upregulation of *Cd146* mRNA (Figure 1G) and protein (Figure 1H) by oxLDL was markedly inhibited by NF-κB inhibitor BAY11-7082. To determine the mechanism of NF-κB-mediated CD146 upregulation, we used a *Cd146* promoter-luciferase reporter assay. We found that *Cd146* promoter activity was robustly enhanced by oxLDL and diminished by NF-κB inhibitor (Figure 1I). Moreover, mutations of the two putative sites abolished the luciferase activity (Figure 1J). In order to further examine whether NF-κB directly binds to these putative sites, we performed a ChIP assay focusing on the p65 subunit of NF-κB. A p65-specific antibody enriched the *Cd146* promoter region in oxLDL-dependent fashion (Figure 1K and 1L). Collectively, these results indicate that the uptake of oxidized lipids of macrophages results in the upregulation of CD146, which is mediated by NF-κB activation.

### CD146 is required for oxLDL-induced macrophage activation

**Figure 2:**
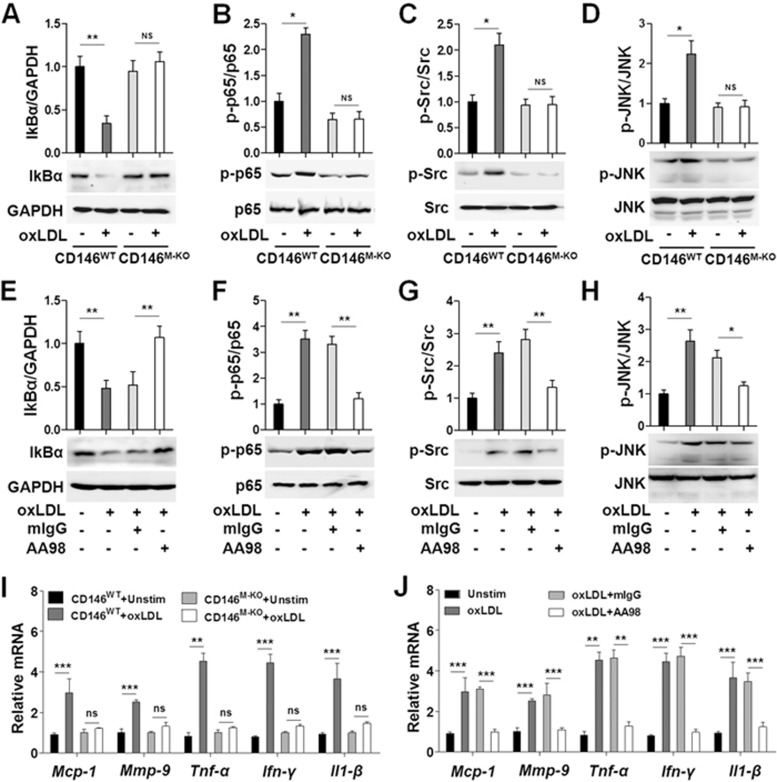
CD146 is required for oxLDL-induced macrophage activation. **(A**-**H)** Western blot analysis of IκBα, phosphorylated (p-) and total NF-κB p65, Src and JNK in oxLDL-stimulated (50 μg/ml) BMDMs isolated from CD146^WT^ or CD146^M-KO^ mice **(A**-**D)** or BMDMs with or without pretreatment with anti-CD146 AA98 (50 μg/ml) **(E**-**H)**. GAPDH was used as a loading control. The upper panel indicated the quantification of CD146 expression. **(I**, **J)** Quantitative real-time (RT) PCR analysis of mRNA levels of the inflammatory factors *Mcp-1*, *Mmp-9*, *Tnf*-α, *Ifn*-γ and *Il1*-β in BMDMs that were treated with oxLDL (50 μg/ml) for 24 h. **(I)** BMDMs were isolated from CD146^WT^ or CD146^M-KO^ mice and stimulated as indicated. **(J)** CD146^WT^ BDMDs were stimulated as indicated in the presence of control mIgG or anti-CD146 AA98 (50 μg/ml). Two-way ANOVA test, ^*^P < 0.05, ^**^P < 0.01, ^***^P < 0.001. The data represent three independent experiments.

To explore the role of macrophagic CD146 during the development of atherosclerosis, we next investigated whether CD146 is involved in oxLDL-induced macrophage activation. We first generated macrophage-specific CD146 knockout mice (CD146^M-KO^) by Cre/LoxP system (Supplementary information, Figure S2). Next, we used oxLDL as a stimulator to evaluate the function of CD146 in the oxLDL-induced signaling cascades in macrophage activation, including NF-κB, Src and JNK^32,33^. The biochemical studies showed that oxLDL induced IκBα/NF-κB, Src and JNK signaling in BMDMs. Importantly, oxLDL-induced signals were blocked in CD146^M-KO^ BMDMs (Figure 2A-2D, and Supplementary information, Figure S3), or by anti-CD146 monoclonal antibody AA98 in CD146^WT^ BMDMs (Figure 2E-2H), indicating that oxLDL activates macrophage in a CD146-dependent manner.

Because the oxLDL-induced macrophage activation is mainly dependent on CD36, we next determined whether CD36 is involved in the process of CD146 on NF-κB activation in response to oxLDL. We observed impaired NF-κB activation in CD36-deficient BMDMs and CD146-deficient BMDMs after stimulation with oxLDL (Supplementary information, Figure S4A). Moreover, knockdown of both CD146 and CD36 had no additional effect on NF-κB activation as compared with CD36 knockdown in RAW264.7 cells (Supplementary information, Figure S4B). These results suggest that the impact of CD146 on NF-κB activation in response to oxLDL is CD36 dependent.

To test whether the influence of CD146 on NF-κB activation is specifically dependent on oxLDL, we repeated the experiments with lipopolysaccharide (LPS) and TNF-α, which can also activate NF-κB. We found that neither CD146 deficiency nor AA98 had any effect on NF-κB activation in response to LPS or TNF-α (Supplementary information, Figure S5), suggesting that the CD146-dependent NF-κB pathway is specifically responsive to oxLDL.

To explore whether CD146 is required for oxLDL-induced transcription of downstream factors, including cytokines, chemokines and matrix metalloproteinases, we performed quantitative real-time PCR to measure the expression levels of a panel of inflammatory factors in response to oxLDL. After treatment of macrophages with oxLDL, increased mRNA levels of *Mcp-1*, *Mmp-9*, *Tnf-α*, *Ifn*-γ and *Il1*-β in macrophages were observed (Figure 2I and 2J). Importantly, macrophages isolated from CD146^M-KO^ mice (Figure 2I) or from CD146^WT^ mice pre-treated with anti-CD146 AA98 (Figure 2J) failed to upregulate these inflammatory genes upon oxLDL treatment, suggesting a critical role of CD146 in oxLDL-induced macrophage activation.

### CD146 promotes oxLDL uptake

**Figure 3:**
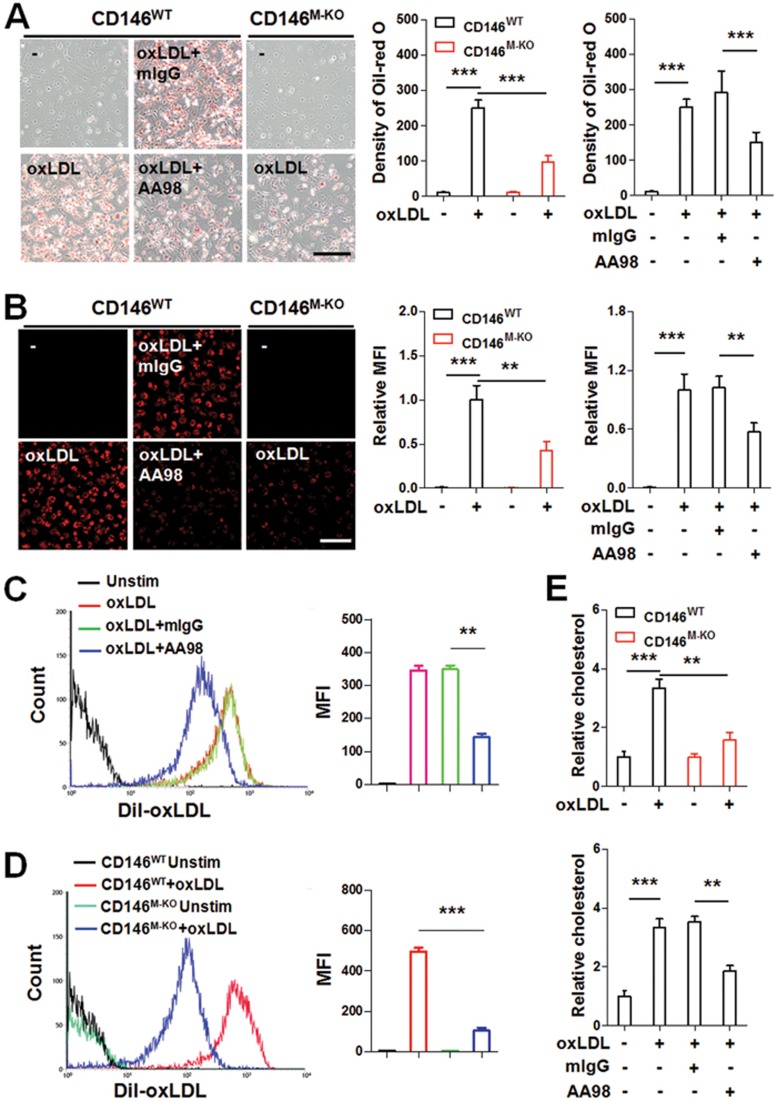
CD146 promotes foam cell formation. **(A)** Lipid uptake was measured by Oil red O staining of CD146^WT^ or CD146^M-KO^ BMDMs that were stimulated with oxLDL (50 μg/ml) for 24 h with or without pretreatment with anti-CD146 AA98 (50 μg/ml). The scale bar is 100 μm. The lower panel shows quantification of oil red O content by means of the Image-Pro Plus software. **(B**-**D)** BMDMs from CD146^WT^ or CD146^M-KO^ mice or BMDMs with or without pretreatment with AA98 (50 μg/ml) were incubated with Dil-oxLDL (50 μg/ml). Dil-oxLDL was detected by either confocal microscopy **(B)** or flow cytometry **(C**, **D)**. Right panel: quantification of the MFI of Dil-oxLDL. **(E)** A biochemical lipid quantitative assay was used to measure total cellular lipids (with two-way ANOVA; ^**^P < 0.01, ^***^P < 0.001). The data represent three independent experiments.

Because CD146 expression on foam cells is involved in oxLDL-induced activation, we then evaluated the possible involvement of CD146 in the uptake of oxLDL by macrophages. Oil red O staining showed that blocking of CD146 with AA98 or with genetic knockdown in macrophages impaired oxLDL uptake (Figure 3A), indicating that CD146 contributes to the uptake of oxLDL by macrophages. Next, we used confocal microscopy and flow cytometry to confirm the role of CD146 in lipid uptake. Dil-labeled oxLDL was used to trace oxLDL uptake. Before detection, cells were washed with ice-cold acid buffer to avoid potential contamination of the cell surface with “sticky” oxLDL. We found that the oxLDL uptake by macrophages was significantly impaired in macrophages from CD146^M-KO^ mice or from CD146^WT^ mice pretreated with anti-CD146 AA98 (Figure 3B-3D). In addition, the measurement of intracellular cholesterol content of macrophages confirmed the facilitating role of CD146 in oxLDL uptake (Figure 3E). All these results indicate that CD146 participates in oxLDL uptake and foam cell formation.

Nevertheless, the reduction in oxLDL uptake by the targeting of CD146 is not due to CD36 downregulation or impaired oxLDL binding to macrophages because neither AA98 engagement nor CD146 deficiency had any effect on CD36 expression (Supplementary information, Figure S6A-S6C) or on the binding of oxLDL to macrophages (Supplementary information, Figure S6D, S6E).

### CD146 interacts with CD36 to facilitate its internalization

**Figure 4:**
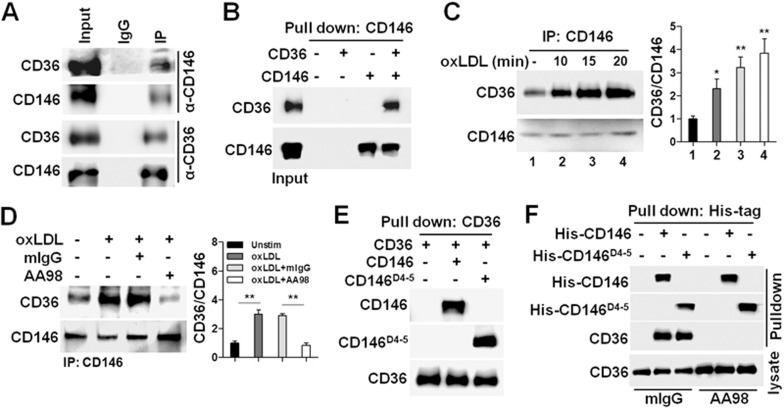
CD146 interacts with CD36. **(A)** Co-IP assay of CD36 and CD146 in BMDMs. CD146 and CD36 from cell lysates were immunoprecipitated with anti-CD146 mAb and anti-CD36, respectively. **(B)** Direct interaction between CD36 and CD146. CD146-ECD was first incubated with CD36-ECD. CD146-ECD was pull down by anti-CD146 ME-9F1, and then precipitated by protein G beads. Bound proteins were subsequently analyzed by western blotting. **(C)** Co-IP assay showed that the CD36 and CD146 interaction was enhanced in the presence of oxLDL in BMDMs. Immunoblot analysis of CD146-precipitated proteins from BMDMs treated with oxLDL (50 μg/ml) for the indicated times. Right panel: quantification of CD36 level relative to CD146. **(D)** Co-IP assay showed that the CD36 and CD146 interaction was decreased by anti-CD146 antibody AA98. Right panel: quantification of CD36 level relative to CD146. **(E)** Direct interaction of CD36/CD146 or CD36/CD146^D4-5^. CD36-ECD was first incubated with CD146-ECD and CD146^D4-5^, respectively. CD36-ECD was pull down by anti-CD36 antibody, and then precipitated by protein G beads. **(F)** AA98 blocked CD36 and CD146 interaction. His-CD146-ECD or His-CD146^D4-5^ was first incubated with BMDM cell lysates in the presence of mIgG or AA98 (50 μg/ml). CD36 proteins bound to His-CD146-ECD or His-CD146^D4-5^ were detected by immunoblot with anti-CD36 antibody. ^*^P < 0.05, ^**^P < 0.01. The data represent three independent experiments.

Because CD36 is the major receptor for oxLDL uptake and subsequently foam cell formation and activation, also based on the finding that both CD146 and CD36 activate NF-κB, Src and JNK signaling in macrophages^28,34^, we hypothesized that CD146 might interact with CD36 to mediate oxLDL uptake. To test this, we first performed coimmunoprecipitation (co-IP) experiments to test whether an interaction exists between CD146 and CD36 in BMDMs. The results showed that both CD36 and CD146 were immunoprecipitated either by anti-CD146 or anti-CD36 antibody, but not control IgG (Figure 4A), suggesting that these two molecules are associated in macrophages. This interaction was confirmed by an *in vitro* pull down assay, in which a direct physical interaction between CD146 and CD36 was detected (Figure 4B). However, CD146 does not interact with oxLDL directly, as shown in the enzyme-linked immunosorbent assay (Supplementary information, Figure S7). Furthermore, we found that the interaction between CD146 and CD36 in macrophage was strengthened by oxLDL in a time-dependent manner (Figure 4C), and was blocked by anti-CD146 AA98 (Figure 4D). Because AA98 specifically recognizes a conformational epitope at C452-C499 disulfide bond within CD146^D4-5^^35^, we expected that the CD146^D4-5^ might bind to CD36. Indeed, CD146^D4-5^ readily bound to CD36 (Figure 4E), and this interaction could be blocked by anti-CD146 AA98 (Figure 4F).

**Figure 5:**
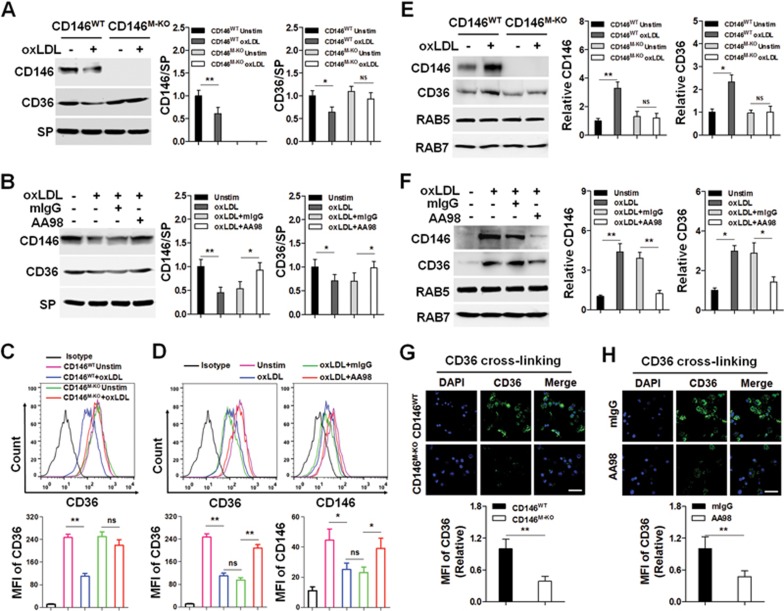
CD146 facilitates CD36 internalization. Western blot **(A**, **B)** and FACS analysis **(C**, **D)** of membrane CD146 and CD36 in oxLDL-stimulated BMDMs isolated from CD146^WT^ or CD146^M-KO^ mice **(A**, **C)** or BMDMs with or without pretreatment with anti-CD146 AA98 (50 μg/ml) **(B**, **D)**. Membrane fractions were immunoblotted with the indicated antibodies. SP (sodium pump) served as a loading control for membrane fractions. Right panel: quantification of CD36 or CD146 levels relative to SP **(A**, **B)**. Bottom panel: quantification of the MFI of CD36 or CD146 **(C**, **D)**. **(E**, **F)** Western blots of CD146 and CD36 in endosomal fractions of BMDMs isolated from CD146^WT^ or CD146^M-KO^ mice **(E)** or BMDMs with or without pretreatment with anti-CD146 AA98 (50 μg/ml) **(F)**. Endosomal fractions were immunoblotted with the indicated antibodies. Right panel: quantification of CD36 or CD146. **(G**, **H)** BMDMs from CD146^WT^ or CD146^M-KO^ mice or BMDMs with or without pretreatment with AA98 (50 μg/ml) were labeled with a CD36-cross-linking antibody. Then, the cells were incubated at 37 °C to cross-link CD36. The cells were then washed with cold acid wash buffer to deplete surface CD36. Confocal microscopy was used to detect and quantify CD36 internalization. Bottom panel: quantification of the MFI of CD36. ^*^P < 0.05, ^**^P< 0.01, ^***^P< 0.001. The data represent three independent experiments.

Because the internalization of CD36 is a crucial step for downstream signal activation in macrophages^23,36,37^, we hypothesized that CD146 may facilitate CD36 internalization after stimulation with oxLDL. Immunoblotting (Figure 5A and 5B) and FACS analysis (Figure 5C and 5D) of plasma membrane fractions of macrophages showed that the internalization of membrane CD36 and CD146 was inhibited in CD146^M-KO^ macrophages as compared with CD146^WT^ macrophages (Figure 5A and 5C), pointing to the impairment of CD36 internalization by the CD146 blockade. Similar results were obtained with AA98-treated WT macrophages (Figure 5B and 5D).

Because binding of oxLDL leads to internalization of the CD36-lipoprotein complex into endosome-like structures^38,39^, we next tested whether CD146 is required for endosomal translocation of CD36 after stimulation with oxLDL. We isolated endosomal fractions (Supplementary information, Figure S8) and analyzed CD36 and CD146 expression by immunoblotting. We found that the oxLDL-induced translocation of CD36 and CD146 to endosomal fractions was inhibited in CD146^M-KO^ macrophages and AA98-treated WT macrophages (Figure 5E and 5F). To confirm this result, we examined their subcellular localization in BMDMs by confocal microscopy. Minimal intracellular localization of CD36 and CD146 was seen in resting cells, but after treatment with oxLDL, CD36 and CD146 shifted to the endosome-like vesicles. In contrast, this process was inhibited in CD146^M-KO^ macrophages and AA98-treated WT macrophages (Supplementary information, Figure S9).

To confirm the function of CD146 in CD36 internalization, we performed a CD36 cross-linking assay. We found that upon binding to a CD36-cross-linking antibody, CD36 internalization was significantly suppressed in CD146-deficient macrophages and in CD146^WT^ macrophages treated with AA98 (Figure 5G and 5H). Collectively, these results suggest that CD146 is involved in lipid uptake by interacting with CD36 and by facilitating the internalization of the receptor-ligand complex.

### CD146 contributes to the retention of macrophages in atheroma

**Figure 6:**
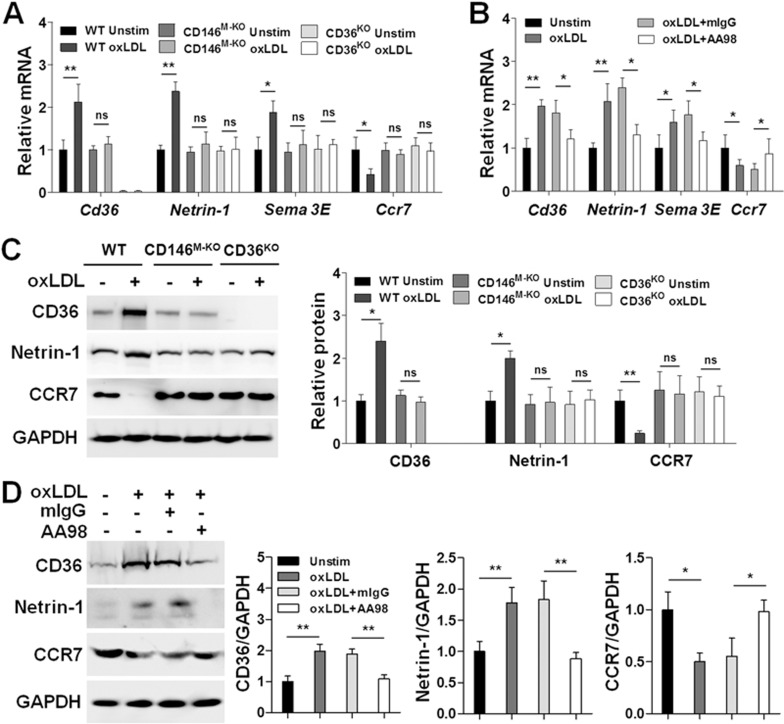
CD146 controls the expression of macrophage migratory factors in response to oxLDL. **(A**, **B)** Quantitative real-time (RT) PCR analysis of mRNA levels of the macrophage migratory factors *Cd36*, *Netrin-1*, *Sema 3E* and *Ccr7* in BMDMs that were incubated with oxLDL (50 μg/ml) for 24 h. **(A)** BMDMs were isolated from WT, CD146^M-KO^ or CD36^KO^ mice and stimulated as indicated; **(B)** WT BDMDs were stimulated as indicated in the presence of control mIgG or anti-CD146 AA98 (50 μg/ml). **(C**, **D)** Western blot analysis of the macrophage migratory factors CD36, Netrin-1 and CCR7 in BMDMs that were treated as indicated. GAPDH was used as a loading control. **(C)** BMDMs were isolated from WT, CD146^M-KO^ or CD36^KO^ mice and stimulated as indicated; **(D)** WT BDMDs were stimulated as indicated in the presence of control mIgG or anti-CD146 AA98 (50 μg/ml). Right panel: quantification of protein expression level relative to GAPDH. ^*^P < 0.05, ^**^P < 0.01. The data represent three independent experiments.

The expression of retention and emigration factors contributes to the retention of macrophages in plaques^7^. Because CD146 promotes oxLDL-induced NF-κB activation, and NF-κB controls the expression of a panel of macrophagic migratory genes, we next determined whether CD146 is required for the expression of macrophage retention factors (CD36, netrin-1 and Sema3E) and emigration factor (CCR7) in response to oxLDL. We found that oxLDL-induced upregulation of macrophage retention factors and downregulation of CCR7 were inhibited by an NF-κB inhibitor (Supplementary information, Figure S10), indicating that NF-κB controls the expression of macrophage migration-related genes. Furthermore, we observed decreased expression of CD36, netrin-1, and Sema3E and increased expression of CCR7 in CD146^M-KO^ and CD36^KO^ macrophages and in AA98-treated WT macrophages (Figure 6), suggesting that CD146 may have an important role in macrophage retention in plaques by partially regulating the expression of migration-related factors.

**Figure 7:**
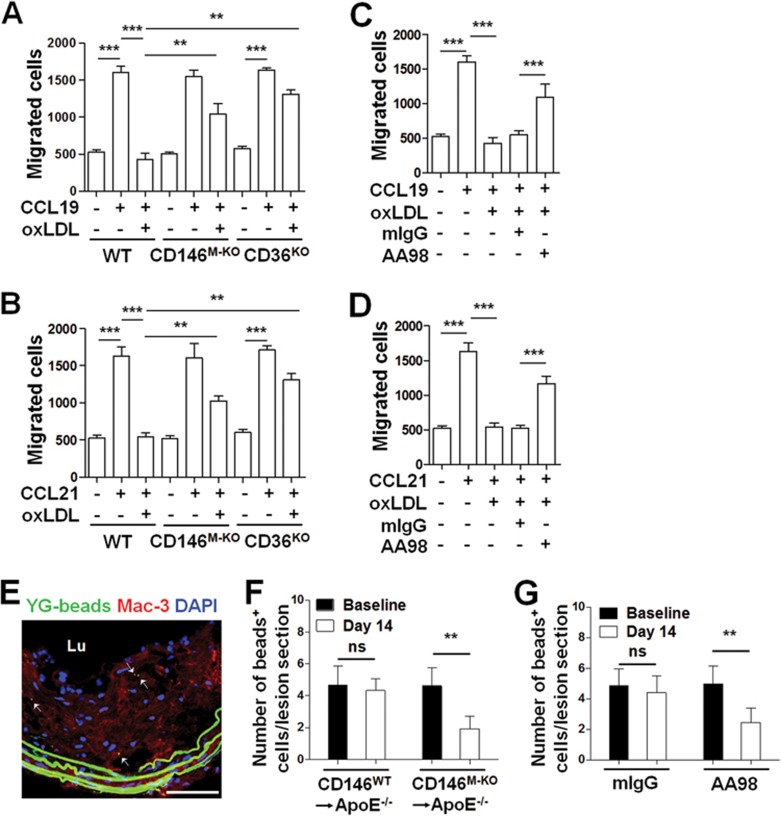
CD146 facilitates the retention of macrophage foam cells. **(A**, **B)** Migration of BMDMs isolated from WT, CD146^M-KO^ or CD36^KO^ mice toward CCL19 **(A)** or CCL21 (500 ng/ml) **(B)** during oxLDL (50 μg/ml) stimulation was measured in a Transwell Boyden chamber (*n* = 5). The number of migrated cells was counted. **(C**, **D)** Migration assay of BMDMs toward CCL19 **(C)** or CCL21 (500 ng/ml) **(D)** during oxLDL (50 μg/ml) stimulation with or without anti-CD146 AA98 (50 μg/ml) using a Transwell Boyden chamber (*n* = 5) (two-way ANOVA test). **(E)** Representative photomicrograph of a lesional section from ApoE^−/−^ mouse at baseline where YG-bead^+^ monocyte-derived cells were observed. Red: Mac-3^+^ cells; blue: DAPI-stained nuclei; green: YG-beads (internal elastic lamina is also green due to autofluorescence). Arrows indicate cells containing fluorescent beads in the lesion section. lu, lumen of aorta. The scale bar is 100 μm. **(F**, **G)** *In vivo* analysis of the recruitment of bead-labeled macrophages (in green) to and retention in atherosclerotic plaques of CD146^WT^→ApoE^−/−^ and CD146^M-KO^→ApoE^−/−^ mice **(F)**, or AA98- or mIgG-treated ApoE^−/−^ mice **(G)** using a monocyte tracking system. The results are presented as beads per area at 10 days (baseline, five mice per group) and 14 days (eight mice per group) after beads injection. At least 20 sections per mouse were analyzed. Two-way ANOVA test. ^*^P < 0.05, ^**^P< 0.01, ^***^P< 0.001. The data represent three independent experiments.

Previous studies showed that the abnormal engulfment of lipids leads to the retention of macrophage in atheroma^18^. We next explored whether CD146 contributes to macrophage retention under hyperlipidemia using the transwell Boyden chamber system. We observed that oxLDL blocked the migration of macrophages induced by chemokine (C-C motif) ligand 19 (CCL19) and CCL21 (Figure 7A-7D), which are the ligands of chemokine receptor CCR7 and facilitate the emigration of macrophage from the atherosclerotic plaques. However, CD146 deficiency, CD36 deficiency (Figure 7A and 7B) and anti-CD146 antibody AA98 (Figure 7C and 7D) restored oxLDL-induced macrophage retention, demonstrating that CD146 is essential for oxLDL-induced macrophage retention.

To further address the role of CD146 in the retention of lipid-loaded macrophages in atherosclerosis plaques, we performed macrophage tracking experiments in CD146^WT^→ApoE^−/−^ and CD146^M-KO^→ApoE^−/−^ bone marrow chimeric mice. In brief, 10 days after microspheres injection (defined here as baseline, corresponding to the time point with the optimal recruitment of labeled monocytes into atherosclerotic plaque and the clearance of the labeled monocytes from the peripheral blood, Supplementary information, Figure S11) and 14 days after injection, the numbers of labeled macrophages in the plaques were measured (Figure 7E). We found that the number of macrophages in CD146^WT^→ApoE^−/−^ and CD146^M-KO^→ApoE^−/−^ plaques was similar to the baseline (Figure 7F), suggesting macrophage migration into the plaques was not affected after CD146 deficiency. However, the number of bead-labeled macrophages in the CD146^M-KO^→ApoE^−/−^ plaques decreased by 60% on day 14; in contrast, CD146^WT^→ApoE^−/−^ plaques showed similar number of beads at baseline and day 14 (Figure 7F), suggesting that fewer macrophages were retained in the lesions in the absence of CD146. These data indicate that CD146 is required for the retention of macrophages in the atherosclerotic plaques. To confirm this, we next performed a macrophage tracking experiment in ApoE^−/−^ mice that were administrated with anti-CD146 or mIgG. We found that in the mIgG-treated group, the numbers of beads-labeled macrophages on day 10 and day 14 were similar, suggesting that most macrophages were retained in the lesions. In contrast, anti-CD146 AA98-treated mice had fewer macrophages (decreasing ∼ 50%) in the plaques on day 14 compared with that at baseline (Figure 7G). These data indicate that CD146 expression on macrophages inhibits their emigration out of the plaques, without affecting their infiltration.

### Targeted deletion of CD146 in macrophages alleviates atherosclerosis

**Figure 8:**
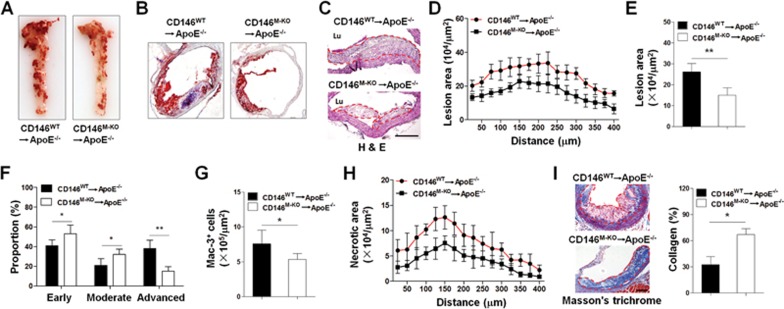
Targeted deletion of CD146 in macrophages leads to lower atherosclerosis burden and less complex plaques. **(A)** Representative images of atherosclerosis in the aorta en face of CD146^WT^→ApoE^−/−^ and CD146^M-KO^→ApoE^−/−^ mice. The aorta was stained with oil red O. **(B)** Representative images of oil red O staining of lesions isolated from CD146^WT^→ApoE^−/−^ and CD146^M-KO^→ApoE^−/−^ mice. **(C)** H&E staining of atherosclerotic lesions isolated from CD146^WT^→ApoE^−/−^ and CD146^M-KO^→ApoE^−/−^ mice (*n* = 8). The dashed lines indicate the lesion borders. The scale bar is 100 μm. **(D)** Lesion area of atherosclerotic plaques of the aortic roots of CD146^WT^→ApoE^−/−^ and CD146^M-KO^→ApoE^−/−^ mice, presented for each genotype across the 400 μm of the aortic root (*n* = 8). **(E)** Quantification of lesion area of aortic plaques isolated from each genotype (*n* = 8). **(F)** The distribution of early, moderate and advanced plaques based on histological staging of the atherosclerotic lesions (*n* = 8). **(G)** Quantification of the number of Mac-3^+^ macrophages in the aortic plaques (*n* = 8, at least 10 sections per mouse). **(H)** Quantification of necrotic core areas of aortic plaques (*n* = 8, at least 20 sections per mouse). **(I)** Masson Trichrome (collagen) staining of aortic plaques (left). Right panel: quantification of staining (*n* = 8, at least 10 sections per mouse). The scale bar is 100 μm. One-way ANOVA test, *P < 0.05, ^**^P < 0.01.

On the basis of above findings, we hypothesized that deletion of CD146 in macrophages might attenuate development of the already established atherosclerosis by inhibiting macrophage retention within the plaques. To test this hypothesis, we generated mice with or without macrophagic CD146 by transferring the CD146^WT^ or CD146^M-KO^ bone marrow cells into lethally irradiated ApoE^−/−^ mice that had been fed a Western diet for 12 weeks. Analysis of plasma revealed no difference in the lipid profile between the two groups. The body weights in the two groups were also unchanged (Supplementary information, Figure S12). Despite similar cholesterol profiles, analysis of the aorta en face and quantification of lesion burden by cross-sectional analysis of the aorta revealed that the CD146^M-KO^→ApoE^−/−^ mice had an atherosclerotic lesion area smaller than that of CD146^WT^→ApoE^−/−^ mice (Figure 8A-8E).

To monitor the progression of atherosclerosis, the lesions were grouped into the following three categories as previously described^40^: lesions with early fatty streaks, moderate lesions with a collagenous cap, and advanced lesions with involvement of the media and increased necrotic area. Our analysis showed that CD146^WT^→ApoE^−/−^ plaques have undergone more severe plaque progression, whereas CD146^M-KO^→ApoE^−/−^ mice had much less advanced plaques (Figure 8F), indicating that macrophagic CD146 promotes the progression of atherosclerotic lesions to more advanced stages. Moreover, staining of Mac-3 confirmed much less macrophages in the plaques of CD146^M-KO^→ApoE^−/−^ mice (Figure 8G). The abundant burden of macrophage foam cells contributed to the instability of atherosclerotic plaque. Analysis of plaque morphology showed that CD146^M-KO^→ApoE^−/−^ mice contained smaller necrotic core (Figure 8H). Moreover, collagen content in the plaques was significantly higher in the CD146^M-KO^→ApoE^−/−^ mice (Figure 8I). Together, these results suggest that CD146 expression in lesional macrophages facilitates atherosclerotic plaque formation and increases plague complexity by enhancing macrophage retention, suggesting macrophagic CD146 may serve as a potential therapeutic target.

### Targeting of CD146 inhibits atherosclerosis

**Figure 9:**
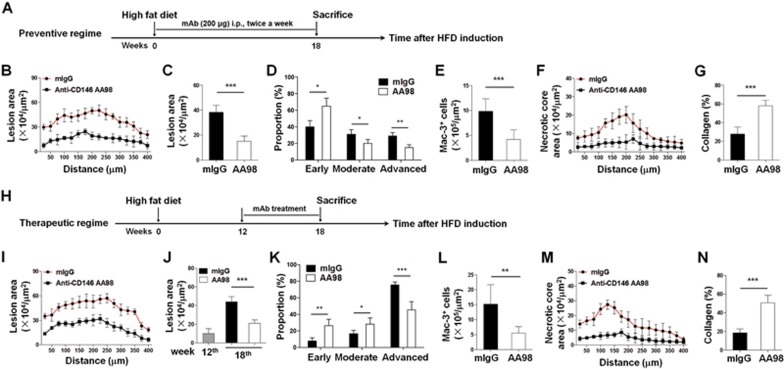
CD146 as a target for atherosclerosis therapy. **(A**-**G)** Preventive targeting of CD146 with antibody reduces atherosclerosis. ApoE^−/−^ mice were preventively injected with mIgG or AA98 when they began a Western diet (eight mice per group). All the quantifications were performed using at least 15 sections per mouse. **(A)** Intervention dosing regimen of CD146-targeted therapy using anti-CD146 AA98 or control IgG in high fat diet (HFD)-induced atherosclerosis. **(B)** Lesion area of plaques of the aortic roots of each group of mice, presented for each group across the 400 μm of the aortic root (*n* = 8). **(C)** Quantification of lesion area of aortic plaques isolated from ApoE^−/−^ mice. **(D)** The distribution of early, moderate and advanced plaques based on histological staging of the atherosclerotic lesions. **(E)** Quantification of immunostained aortic plaques for macrophages using the macrophage marker Mac-3. **(F)** Quantification of necrotic core areas of aortic plaques. **(G)** Quantification of Masson's trichrome (collagen) staining of aortic plaques. **(H**-**N)** Therapeutic targeting of CD146 with antibody reduces atherosclerosis (five mice per group). All the quantifications were performed using at least 15 sections per mouse. **(H)** Intervention dosing regimen of CD146-targeted therapy using anti-CD146 AA98 or control IgG in HFD-induced atherosclerosis. ApoE^−/−^ mice were fed a HFD for 12 weeks before antibody treatment for additional 6 weeks, the time at which the mice were sacrificed. **(I)** Lesion area of atherosclerotic plaques of the aortic roots of each group of mice, presented for each group across the 400 μm of the aortic root (*n* = 5). **(J)** Quantification of lesion area of aortic plaques isolated from ApoE^−/−^ mice. **(K)** The distribution of early, moderate and advanced plaques based on histological staging of the atherosclerotic lesions. **(L)** Quantification of immunostained aortic plaques for macrophages using the macrophage marker Mac-3. **(M)** Quantification of necrotic core areas of aortic plaques. **(N)** Quantification of Masson Trichrome (collagen) staining of aortic plaques. The scale bar is 100 μm. One-way ANOVA test, ^*^P< 0.05, ^**^P< 0.01, ^***^P< 0.001.

Because CD146 contributes to oxLDL uptake and retention of macrophage foam cells, and targeting macrophagic CD146 with the AA98 antibody (which recognizes murine CD146, Supplementary information, Figure S13) inhibited lipid uptake and promoted emigration of macrophages, macrophagic CD146 may serve as a potential therapeutic target for atherosclerosis. We next examined the preventive and therapeutic effects of AA98 in treating atherosclerosis. First, we fed ApoE^−/−^ mice with a Western diet for 18 weeks and simultaneously treated them with AA98 or control IgG. The mice were then sacrificed, and development of atherosclerotic lesions was quantified by performing a histological analysis of consecutive sections (Figure 9A). Interestingly, compared with the control mIgG-treated mice, the plaque size in the AA98-treated mice was significantly reduced (Figure 9B and 9C). Histological characterization analysis of the plaques revealed that the AA98-treated mice had fewer advanced (15%) and earlier stage (65%) lesions than the control group (Figure 9D). Mac-3 staining showed that fewer macrophages were observed in the lesions of AA98-treated mice than those of the mIgG-treated mice (Figure 9E). Moreover, we observed smaller necrotic core (Figure 9F) and higher collagen content (Figure 9G) in the plaques of the AA98-treated mice. However, the serum level of triglyceride, cholesterol, HDL, LDL, FFA and oxLDL, as well as the body weight was similar between the two groups (Supplementary information, Figure S14A and S14B), suggesting that targeting of CD146 with antibody inhibited atherosclerosis development without affecting serum cholesterol.

To test whether CD146 could serve as a therapeutic target for established atherosclerotic plaques, we treated ApoE^−/−^ mice that had been fed a cholesterol-rich diet for 12 weeks (with apparent plaques as shown in Figure 9J) with either anti-CD146 antibody AA98 or a control antibody for an additional 6 weeks (Figure 9H). Compared with the control group, we observed smaller plaque size (Figure 9I and 9J) as well as much less plaque complexity (Figure 9K), less macrophage accumulation (Figure 9L), smaller necrotic core (Figure 9M) and higher collagen content (Figure 9N) in the plaques of the AA98-treated mice. However, the lipid profiles and the body weight were not changed between the two groups (Supplementary information, Figure S14C and S14D). Together, these results suggest that targeting of CD146 might be a feasible therapeutic strategy for inhibiting the development of atherosclerosis through promoting the emigration of macrophages from the plaque.

## Discussion

It is well known that atherosclerosis is an immune system-mediated, nonresolving inflammatory disease. The formation and retention of cholesterol-engorged macrophages in atheroma exacerbates the disease and fuels the development of vulnerable plaques, causing clinical events^2,3^. Therefore, strategies aimed to diminish the formation of macrophage foam cells and promoting their emigration can slow the lesion progression, which may have a potential as an adjunctive treatment for standard lipid-lowering therapies^6,41^. Nonetheless, the mechanisms that regulate foam cell formation and the signals that trap macrophages within (or guide macrophages to exit) plaques for the most part remain poorly understood^6,42^. Here, we report that CD146 participates in the pathogenesis of atherosclerosis by driving the formation and retention of macrophage foam cells in atherosclerotic plaques, as supported by several lines of evidence: (1) CD146 is required for oxLDL uptake and foam cell formation because of the promotion of CD36 internalization; (2) CD146 contributes to oxLDL-induced activation of NF-κB, Src, and JNK and to the production of proinflammatory factors; (3) CD146 promotes the expression of retention factors while inhibiting the expression of emigration factors; (4) CD146 contributes to oxLDL-induced macrophage retention in plaques; (5) deletion of CD146 in macrophages or targeting of CD146 with an antibody promotes macrophage emigration out of the plaque and reduces macrophage content of the plaque. These findings reveal a causative role of CD146 in the activation and retention of lipid-loaded macrophages in plaques and suggest that CD146 is a novel therapeutic target in atherosclerosis.

The accumulation of lipids is essential for the differentiation of macrophages and the formation of foam cells. Scavenger receptors (ScRs) and toll-like receptors (TLRs) expressed on macrophages mediate foam cell formation^4,7^. The interaction between oxLDL and CD36 on macrophages triggers signaling responses that are both pro-inflammatory and pro-atherogenic, leading to ligand internalization, lipid accumulation, foam cell formation and the inhibition of cell emigration^11,43^. Foam cells in the atheroma are essential to the instability of plaques by releasing several factors, including matrix metalloproteinases, NO and endothelin^44,45,46^. As key players in atherosclerosis, foam cells have a broad network of interacting genes and proteins to promote atherogenesis^4,47^. Many proteins that are differentially expressed in foam cells are involved in lipid binding, cytoskeletal regulation and vesicle-mediated transport. Inflammation-associated proteins are also differentially expressed in desmosterol-loaded foam cells^48^. However, despite this wealth of knowledge, the mechanisms underlying foam cell generation remain poorly understood. Our study reveals a novel mechanism underlying hyperlipidemia-induced formation of macrophage foam cells. We first identified CD146 as a novel regulator of CD36 upregulation during stimulation with oxLDL. Some studies suggest that CD36 upregulation by oxLDL is mediated by PPARγ^49^. In line with this observation, we found that the impact of CD146-induced CD36 upregulation is also PPARγ dependent (Supplementary information, Figure S15) although the detailed mechanism is unknown. In addition, CD146 is a novel interacting partner of CD36 and promotes the receptor internalization and lipid uptake. The region of CD146 that interacts with CD36 was found in CD146^D4-5^, which is specifically recognized by anti-CD146 AA98^35^. This newly identified interaction between CD146 and CD36 facilitates the uptake of lipids and foam cell formation and partially explains why anti-CD146 is effective at inhibiting CD146- and CD36-mediated foam cell formation and retention within the plaque.

Mounting evidence has shown that various factors contribute to the vulnerability of atherosclerotic plaques. In particular, macrophage foam cells are the major players that trigger plaque instability^5,6^. Lipids accumulated in macrophages can be transported out of the cells via ATP-binding cassette lipid transporters under normal lipidemia conditions^50^; however, lipid metabolism of macrophages in atherosclerotic plaques is impaired due to a reduction in lipid transporters. The overload lipids in macrophages block macrophage emigration from the plaques and result in apoptosis and secondary necrosis, which in turn exacerbates atherosclerosis^7,51^. Inhibiting the uptake of lipids and/or promoting the emigration of macrophages from the plaques will improve the clinical outcome for patients with advanced atherosclerotic lesions. The normalization of serum HDL-C level decreases CD68-positive macrophages and induces the expression of chemokine receptor CCR7, which in turn facilitates the emigration of macrophages from the plaque^14^. Moreover, depleting the ScR SR-A or CD36 slows the progression of atherosclerosis^52,53^. Recent studies examined the mechanism of macrophage retention in the plaques and found that the neuroimmune guidance cues netrin-1 and Semaphorin 3E are pro-atherogenic factors that promote the retention of macrophages in the arterial wall^17,18,19^. In our study, we reveal a novel regulator CD146 in macrophage retention by regulating both foam cell formation and inhibiting emigration from plaques. We also provide evidence that CD146 may serve as a viable target for attenuating non-resolving inflammation.

A growing body of evidence suggests that the recognition of oxLDL by CD36 triggers a cascade of signals that facilitate macrophage activation and production of proinflammatory factors^12^. Although these downstream signals have been identified, the mechanisms facilitating ligand-induced CD36 internalization are poorly understood. Various studies are suggestive of an emerging paradigm in which CD36 acts together with other cell surface molecules to bridge oxLDL recognition and initiation of signaling responses. The recognition of oxLDL triggers assembly of a heterotrimeric complex composed of CD36 and TLRs 4 and 6, thus leading to stimulation of sterile inflammation^37^. Recently, it was reported that the oxLDL-CD36 complex recruits the Na^+^/K^+^-ATPase-Lyn complex in macrophages and thereby promotes atherosclerosis^11^. Moreover, CD36 associates with integrins and tetraspans, which facilitate its internalization^36^. These studies suggest that formation of the CD36 signalosome is required for the activation of a number of pathways and have led to the notion that CD36 functions in heteromultimeric signaling complexes. Our study reveals a novel component of this heteromultimeric signalosome, which functions at the cell membrane. The mechanistic role of CD146 was found to mediate oxLDL internalization without effecting oxLDL binding to macrophages as evidenced by a series of lipid uptake experiments. This uptake process involves the co-internalization of CD36 with CD146 from the plasma membrane to endosomal-like vesicles. Nonetheless, how CD146 coordinates these heteromultimeric components while driving CD36/oxLDL internalization and endosomal translocation remains unclear.

Studies by multiple groups point to a CD36-dependent mechanism of macrophage trapping in atheroma. The major downstream signals of CD36 include Src, FAK, JNK and NF-κB, each with distinct functions^23^. Specifically, activation of Src plus JNK or Src plus vav drives oxLDL uptake^33^; simultaneous activation of Src, ROS and FAK promotes macrophage spreading^12^; CD36 participates in the activation of NF-κB and NLRP3 inflammasome as well as in the production of proinflammatory cytokines^54^. In our study, we found that by driving oxLDL/CD36 internalization, CD146 controls oxLDL-induced Src, JNK and NF-κB activation. Therefore, in coordination with CD36, macrophagic CD146 acts as an upstream regulator to trigger the signaling cascades that mediate the uptake of oxLDL, cytokine production and macrophage trapping. The net effect is the proatherosclerotic inflammatory process.

It has been reported that CD146 expression and activation of NF-κB are regulated reciprocally. In endothelial cells, expression of CD146 is under the control of VEGF and TNF-α through NF-κB transactivation^27,55^. Furthermore, as a coreceptor of VEGFR2, CD146 is one of the key components of the VEGFR2 signalosome and facilitates VEGF-induced NF-κB activation and expression of proinflammatory factors^35,56^. This reciprocal regulation between CD146 and NF-κB has also been observed in macrophages during hyperlipidemia. We found that during atherosclerosis, macrophagic CD146 expression was significantly increased by oxLDL-induced NF-κB activation. The upregulated CD146 performs an important function in NF-κB signaling by regulating the CD36 receptor-ligand complex. This phenomenon in turn results in the accumulation of CD146, creating a positive feedback loop crucial for establishing a proinflammatory microenvironment. Moreover, we also found that the CD146 knockout or blockade with an antibody inhibited CD146-mediated oxLDL, CD36, and NF-κB signaling and expression of macrophage retention factors, such as netrin-1, Sema3E and CD36 while promoting the expression of emigration factor CCR7 both *in vitro* and *in vivo* (Supplementary information, Figure S16). These data indicate that CD146 may be upstream of these migratory factors. Therefore, our study has uncovered a novel feedback loop orchestrated by CD146, CD36 and NF-κB during oxLDL uptake and macrophage activation and retention.

In our study, we demonstrated that the role of macrophagic CD146 in atherosclerosis is mainly through promotion of CD36-dependent formation of foam cells and their retention in the plaques. We found that one of the signals downstream of CD146 in macrophages is NF-κB. We showed that the influence of CD146 on NF-κB activation in response to oxLDL is CD36 dependent. However, there might be CD36-independent pathways that are responsible for anti-CD146. Except for CD36, we observed that CD146 regulates the expression of several macrophage retention-related factors, including netrin-1 and CCR-7, which might contribute to macrophage retention. Moreover, the non-canonical Wnt5a has recently emerged as an atherogenic factor^57^, and CD146 functions as a novel receptor for Wnt5a^58^. These observations suggest that CD146 deficiency or anti-CD146 might not be fully dependent on the CD36-dependent pathway. However, whether these CD36-independent pathways are responsible for the reduction of atherosclerosis in CD146 deficiency or anti-CD146 treatment would require further research.

With regard to the anti-CD146 therapy for atherosclerosis in mice, there may be potential side effects. It has been reported that CD146 expression is detectable in the vascular wall (including endothelial cells of small vessels, smooth muscle cells and pericytes), in small subsets of peripheral T lymphocytes, and in mesenchymal stem cells in normal tissues^28^. Because CD146 has been implicated in vascular development, stem cell differentiation, cell migration and T cell activation, one might expect adverse effects of anti-CD146 therapy. Although we previously reported that anti-CD146 AA98 does not affect T lymphocyte proliferation or activation in mice^59^, whether anti-CD146 treatment can have adverse effects by targeting vascular wall or mesenchymal stem cells is an interesting question that is waiting to be addressed in our future studies.

In this study, we employed a bone marrow transplantation assay to reveal that the role of CD146 in the preexisting atherosclerotic lesions was partially due to its function on macrophage emigration. It has been reported that macrophages emigrate from plaques during disease regression, but not progression^10,60^. Moreover, normalization of plasma lipids by transferring of ApoE^+^ bone marrow promoted the regression of atherosclerosis and led to the emigration of intra-plaque macrophages out of lesions^4,61^. Although we observed reduced levels of plasma lipids in both groups due to transplantation of ApoE^+^ bone marrow, macrophagic CD146 deficiency showed no effect on plasma lipid profiles. Therefore, the differences in development of the established plaques between the two groups might partially resulte from enhanced emigration of CD146-null macrophages from the regressive lesions independent of lowering plasma cholesterol levels.

In this study, we found that the production of netrin-1 by macrophages is tightly controlled by the CD146-CD36-NF-κB axis, suggesting that CD146 and CD36 act upstream of netrin-1 when inhibiting macrophage migration. In fact, the role of netrin-1 in cell migration is highly complex, in which studies have revealed a dual role of netrin-1 in cell migration. By interacting with various receptors, netrin-1 can act as a promigratory or antimigratory factor. The function of netrin-1 depends on its concentration and the affected receptors^62^. At high concentrations, netrin-1 inhibits migration through its receptor called UNC5B, but at relatively low concentrations, promotes migration through CD146^63^. During atherosclerosis, plaque macrophages secrete netrin-1 to inactivate their migration via its receptor UNC5B^18^. It seems that the netrin-1-UNC5B complex triggers signals that counteract netrin-1-CD146-related promigratory signals, thus inhibiting cell migration in this context. Therefore, we propose that CD146 promotes atherosclerosis by CD36-dependent production of retention factors including netrin-1. Subsequently, netrin-1 inhibits macrophage migration via its receptor UNC5B.

In summary, we report for the first time that CD146 has a pro-atherogenic role by facilitating the formation and retention of macrophage foam cells in atherosclerotic plaques. Our findings highlight the potential of this adhesion receptor as a therapeutic target for atherosclerosis treatment.

## Materials and Methods

### Antibodies and reagents

Anti-CD146 antibodies, including mouse anti-CD146 mAb AA98 and AA4, were described previously^59,64^. AA98 was used for functional assays, AA4 for immunohistochemistry (paraffin-embedded) and rat anti-mouse CD146 (clone: ME-9F1) for western blot and pull-down assays.

Other antibodies were used in this study: anti-NF-κB p65 (cat. #3031s, Cell Signaling); anti-IκBα (cat. #4812, Cell Signaling), p-p65 (cat. #3031s, Cell Signaling); anti-GAPDH (cat. #ab8245, Abcam); anti-JNK (cat. #9252, Cell Signaling), anti-p-JNK (cat. #4668, Cell Signaling), Src (cat. #2109, Cell Signaling), p-Src (cat. #2101, Cell Signaling); anti-LAMP-1 (cat. #ab24170, Abcam); anti-RAB5 (cat. #ab18211, Abcam); anti-RAB7 (cat. #ab50533, Abcam); anti-toll-like receptor 4 (cat. #14358, Cell Signaling); anti-toll-like receptor 6 (cat. #12717, Cell Signaling); anti-human CD68 (cat. #14-0689-80, eBioscience); anti-mouse CD36 (cat. #5777-1, Epitomics); anti-CD36 IgA (cat. #ab23680, Abcam); anti-netrin-1 (cat. #ab126729, Abcam); anti-CCR7 (cat. #2059, Epitomics); anti-mouse CD146 (cat. #134701, Clone 9F1, Biolegend); anti-mouse Mac-3 (cat. #108502, Biolegend); APC-conjugated anti-mouse F4/80 (cat. #M100F1-11A, SungeneBiotech); PerCP-Cy5.5-conjugated anti-mouse CD11b (cat. #550993, BD Pharmingen); isotype-matched control antibody mIgG (Sigma-Aldrich); and horseradish peroxidase-conjugated anti-mouse and anti-rabbit secondary antibodies (GE Healthcare).

Recombined murine CD36-His protein (cat. #50422-M08H) and murine CD146-His protein (cat. #50794-M08H) were obtained from Sino Biological Inc. Recombined murine chemokines CCL19 (cat. #250-27B) and CCL21 (cat. #250-13) were available from PeproTech. LDL, Cu^2+^-oxLDL, AcLDL and Dil-labeled oxLDL (Dil-oxLDL) were purchased from Peking Union-Biology Co., Ltd. Fluoresbrite Microparticles (cat. #17154) were purchased from Polysciences Inc. T0070907 and Troglitazone was purchased from Sigma.

### Mice

C57BL/6J and apolipoprotein E-deficient (ApoE^−/−^) mice were obtained from the Department of Laboratory Animal Science, Peking University Health Science Center. CD36^−/−^ mice were obtained from the Nanjing Biomedical Research Institute of Nanjing University.

For generating macrophage-specific CD146 knockout mice (CD146^M-KO^, Lyz2^cre/+^CD146^floxed/floxed^), Lyz2^cre/+^CD146^+/+^ mice (obtained from the Nanjing Biomedical Research Institute of Nanjing University) were firstly crossed with Lyz2^+/+^CD146^floxed/floxed^ mice (obtained from the Nanjing Biomedical Research Institute of Nanjing University, backcrossed onto a C57BL/6J background for a minimum of nine generations). The F1 Lyz2^cre/+^CD146^floxed/+^ genotype was backcrossed with Lyz2^+/+^CD146^floxed/floxed^ mice to generate Lyz2^cre/+^CD146^floxed/floxed^ mice, which we call CD146^M-KO^ mice. Lyz2^+/+^CD146^floxed/floxed^ mice (which we call WT mice here) were used as controls. All genotypes were confirmed by PCR analysis. CD146 deficiency in macrophages was confirmed by immunoblotting (Supplementary information, Figure S2). All mice were maintained in a pathogen-free facility. All animal experiments were performed in compliance with the guidelines for the care and use of laboratory animals and were approved by the institutional biomedical research ethics committee of the Institute of Biophysics, Chinese Academy of Sciences.

### Atherosclerosis model induction, treatment and atherosclerotic analysis

For atherosclerosis induction, 8-week-old ApoE^−/−^ mice were fed a high fat Western diet (Teklad Adjusted Calories 88137: 21% (wt/wt) fat; 0.15% (wt/wt) cholesterol; 19.5% (wt/wt) casein; no sodium cholate). For prevention, the anti-CD146 monoclonal antibody AA98 or control mIgG (200 μg per mouse, twice a week) was injected i.p. on the first week of WD for 18 weeks (eight mice per group). For therapy, the antibodies were injected (200 μg per mouse, twice a week) on the 12th week of the WD, and the injections were continued for another 6 weeks (five mice per group). At the end of the experiments, the mice were sacrificed and perfused with PBS, after which the aortic arteries were dissected and either frozen in OCT or fixed with 4% paraformaldehyde (PFA) for immunohistochemistry. Classification of aortic plaques was carried out according to severity as early, moderate and advanced, as described before^40^. For cross-sectional analysis of lesion areas in the aortic root, sections (8 μm in thickness) throughout the aortic sinus (400 μm) were obtained for analysis and quantified with ImageJ software. Necrotic areas, defined as acellular and anuclear white areas, were analyzed from hematoxylin- and eosin-stained sections and quantified with ImageJ software.

### Clinical sample collection

Atherosclerosis patients from Beijing Anzhen Hospital were selected on a clinical basis. Written informed consent was obtained from each patient, and prior to sample collection, ethics approval was obtained from the Ethics Committee of the Anzhen Hospital and the Institute of Biophysics, Chinese Academy of Sciences. Human carotid artery segments from atherosclerosis lesions as determined by carotid ultrasound were obtained from carotid endarterectomies, fixed in neutral buffered formalin, and then embedded in paraffin for immunohistochemistry.

### Dural luciferase reporter assay

The pGL3 firefly luciferase plasmid containing a 2-kb CD146 promoter region was constructed as previously described^27^. The mutants of putative NF-κB binding sites of CD146 promoter regions were generated by PCR and confirmed by sequencing. The pGL3 vectors containing wild-type or mutant CD146 promoter were then transfected into HEK293 cells, together with pRLTK containing the Renilla luciferase reporter gene and the control empty plasmid. Twelve hours after transfection, cells were treated with oxLDL (50 μg/ml) for 24 h. Firefly and Renilla luciferase activities were then measured with the Dual-Luc Assay Kit (Promega).

### ChIP and PCR

Specific protein-DNA interactions were examined by ChIP followed by qPCR (Chromatin Immunoprecipitation Assay Kit, Millipore). Protein-DNA crosslink was achieved by fixation with 1% formaldehyde for 10 min at room temperature. DNA-protein complexes from 2 × 10^6^ cells were sheared to lengths between 200 and 500 base pairs by sonicator. The pre-cleared fragments were incubated with 10 μg of p65 specific antibody, or IgG (as a negative control) overnight, followed by immunoprecipitation by Protein A. The crosslink was reversed by heating at 65 °C overnight, followed by Proteinase K digestion at 45 °C for 2 h. DNA was then recovered with QIAquick PCR purification kit (Qiagen) for qPCR to prove affinity against CD146 promoter region. The primers for putative NF-κB binding sites were: NF-621 sense, 5′-GACTTGCAGGAGCTTGCGTTTG-3′ and antisense, 5′-CGATTGCACCACTGCCGCT-3′ and NF-420 sense, 5′-GCGGCAGCGGCAGTGGTG-3′ and antisense, 5′-GGTAGTGACAGGTGTCTCGGG-3′. GAPDH sense, 5′-CGGAGTCAACGGATTTGGTCGTAT-3′, antisense 5′-AGCCTTCTCCATGGTGGTGAAGAC-3′ was used as control. The PCR products were separated on 1.5% agarose gels.

### Macrophage isolation and induction

Peritoneal macrophages were harvested from peritoneal lavage of mouse (WT mice and CD146^M-KO^ mice) 7 days after an i.p. injection of 100% paraffin oil. The cells were washed in PBS and were cultured overnight in DMEM (with 10% FBS) and stimulated with oxLDL (50 μg/ml). Bone myeloid-derived macrophages (BMDMs) were collected from the long bones; after the red blood cells were lysed, the BMDMs were plated on 10-cm culture dishes and cultured with DMEM (with 15% FBS) supplemented with 20% (vol/vol) L929 conditioned medium for 8 days.

### Real-time PCR

Total RNA was extracted with Trizol reagent (Invitrogen). RNA (2 μg) was reverse transcribed into cDNA by random primers. Real-time PCR analysis was performed on a Corbett 6200 using SYBR Green PCR mix (Toyobo Co., Osaka, Japan). Threshold cycle (Ct) values of GAPDH were subtracted from Ct values of the genes of interest (ΔCt). All primers used were synthesized by Invitrogen using the sequences listed in Supplementary information, Table S1.

### Migration assays

Macrophage chemotaxis was measured in a 96-well Boyden chamber containing a filter (Corning Costar) with a pore size of 8 μm. The same number (1 × 10^4^) of BMDMs was grown with DMEM (with 5% FBS) in the upper chamber. Mouse IgG or anti-CD146 mAb AA98 (50 μg/ml) was added 1 h before migration, and oxLDL (50 μg/ml) was added to the upper chamber for stimulation. The cell migration was initiated by filling the lower chamber with DMEM (with 10% FBS) supplemented with CCL19 or CCL21 (500 ng/ml) and lasted for 12 h. The migrated cells to the lower membrane was stained with crystal violet and counted under a microscope.

### Oil red O staining

The BMDMs were incubated with oxLDL for 24 h. The cells were fixed with 4% PFA and then washed with PBS and stained with oil red O for 20 min at 37 °C. The cell morphology was observed using an optical microscope equipped with an imaging system. The quantification of oil red O content was measured by software Image-Pro plus.

### Intracellular cholesterol measurement in macrophage-derived foam cell

Mice bone marrow-derived macrophage in six-well plates was treated with oxLDL (50 μg/ml) for 24 h. After the cells were washed twice with PBS, the intracellular cholesterol was distracted by added 0.5 ml hexane : isopropanol (3:2), collected in vials and dried in fume hood. The Amplex Red Cholesterol Assay Kit (cat. #A12216, Invitrogen) was used to detect the total cholesterol in foam cells. The protein content was detected using the BCA assay kit from Pierce.

### Dil-oxLDL uptake assay

Dil-labeled oxLDL was used to trace the oxLDL uptake. Mice bone marrow-derived macrophages were treated with oxLDL (50 μg/ml) for 24 h, and then washed twice with cold PBS before staining. Cold acid washing buffer (0.5 M glacial acetic acid, 150 mM sodium chloride, pH 2.5) was used to wash surface adherent ox-LDL. Cells were then fixed with 4% PFA. The cells were visualized using a confocal laser scanning microscope (Olympus, Tokyo, Japan). The mean fluorescent intensity was measured by software Olympus Fluoview Viewer. Alternatively, the cells were detached from the plate, and cell surface markers CD11b and F4/80 were stained on ice for 30 min. After two times of wash by FACS washing buffer (2% FBS in PBS), samples were analyzed by FACS. Data were further quantified by software FlowJo.

### Detection of cell surface CD36 expression by FACS and immunoblot analysis

Mouse BMDMs were incubated with or without oxLDL (50 μg/ml) for 15 min in the presence of mIgG or anti-CD146 AA98 (50 μg/ml). The cells were then harvested and stained with anti-CD36 antibodies at 4 °C before FACS analysis. Alternatively, the plasma membrane proteins were isolated by means of the Minute Plasma Membrane Protein Isolation Kit (cat. #SM-005, Invent Biotechnologies, Inc.), which offers rapid isolation of plasma membrane proteins from cultured cells without contamination with organelles and other membranous fractions. The fraction containing plasma membrane proteins was then prepared for immunoblot analysis.

### The CD36 cross-linking and internalization assay

CD36 cross-linking and internalization were performed as described previously^36,65^. Macrophages were serum starved for 5 h before two washes with ice-cold RPMI 1640 to prevent endocytosis during labeling. Then, the cells were incubated with anti-CD36 IgA (1:500) for 10 min on ice. After two washes with cold RPMI 1640, the cells were incubated with a FITC-conjugated anti-mouse IgA antibody for another 10 min to cross-link CD36. Next, the macrophages were transferred to a 37 °C incubator, with incubation for 30 min in RPMI 1640 to allow for internalization of CD36. Internalization was then stopped by addition of cold RPMI 1640. For detection of intracellular CD36, surface-bound antibodies that were not internalized were removed by a 2-min acid wash with cold acid wash buffer (0.5 M glacial acetic acid, 150 mM sodium chloride, pH 2.5). The cells were then fixed for 15 min in 4% PFA on ice. Z-stacks (0.5 μm/slice) of the labeled cells were obtained using a confocal laser scanning microscope (Olympus, Tokyo, Japan).

### Endosome isolation

Endosomes were isolated by density gradient centrifugation as previously described^66^. Briefly, BMDMs were homogenized using a glass tissue grinder in a buffer consisting of 250 mM glucose, 3 mM imidazole, and 1 mM EDTA, pH 7.4 and protease inhibitors. Next, the postnuclear supernatant was purified by sequential centrifugation of the total cell homogenates for 10 min at 700× *g* and 30 min at 17 000× *g*. Endosomes were isolated from the postnuclear supernatant by differential centrifugation in a discontinuous sucrose gradient (consisting of three steps: 8%, 35% and 42% sucrose).

### Immunoprecipitation

Cells were lysed with immunoprecipitation buffer (150 mM NaCl, 50 mM Tris, pH 8.0, 0.1% SDS, 0.5% deoxycholate, 1% NP-40, 1 mM phenylmethanesulfonyl fluoride and Protease Inhibitor Cocktails) for 0.5 h on ice. After centrifugation, the soluble supernatants were pre-cleared with protein G PLUS-Agarose (Santa Cruz Biotechnology Inc.). Samples were then immunoprecipitated with specific antibodies and 25 μl of protein G-agarose beads. Protein G-bound immunocomplexes were extensively washed with washing buffer (150 mM NaCl, 50 mM Tris, pH 8.0, 0.1% SDS, 0.5% deoxycholate, 0.1% NP-40, 1 mM phenylmethanesulfonyl fluoride and Protease Inhibitor Cocktails) and boiled in sample loading buffer for SDS-polyacrylamide gel electrophoresis. The immunoprecipitated proteins were detected by immunoblotting.

### Immunoblot analysis

For immunoblotting, proteins were separated with 10% SDS-polyacrylamide gel electrophoresis and then transferred to a nitrocellulose membrane. The membranes were then blocked with 5% non-fat milk in phosphate-buffered saline with 0.1% Tween-20 for 1 h, incubated for 1 h with primary antibodies and then probed with horseradish peroxidase-conjugated anti-mouse, anti-rat or anti-rabbit secondary antibodies. All immunoblots were carried out using chemiluminescence reagent (Pierce) and exposed to X-ray films (Kodak, New York, NY, USA). Densitometry was analyzed with ImageJ software.

### In vitro pull-down assays

In all, 0.15 μg His-tagged CD146 or CD146^D4-5^ was incubated with CD36 protein for 1 h in the presence or absence of anti-CD146 mAbs. Proteins bound to the beads were then boiled in sample loading buffer and subjected to immunoblotting.

### Enzyme-linked immunosorbent assay-based binding curve

oxLDL (5 μg/ml) was coated on 96-well plates overnight at 4 °C. About 0.625-20 μg/ml of His-CD146 or His-CD36 protein were then added into the oxLDL-coated wells and incubated for 3 h at room temperature. Mouse anti-His primary antibody, horseradish peroxidase-conjugated goat-anti-mouse secondary antibody and horseradish peroxidase substrate were then sequentially added to the wells and incubated for 1 h. Absorbance values were then read at OD450.

### Immunohistochemical and immunofluorescence staining

Atherosclerotic plaque morphology and collagen content was examined in 8-μm-thick sections stained with hematoxylin and eosin and Masson's trichrome, respectively. For 3,3′-diaminobenzidine staining, paraffin-embedded tissue sections were deparaffinized and stained first with an antibody specific for CD146 (AA4), followed by a biotin-conjugated secondary antibody (1:1 000), and then by HRP-conjugated streptavidin (Dianova, Rodeo, CA). Macrophages were identified using anti-Mac-3 (a marker for murine macrophages). The sections were counterstained with hematoxylin. The number of macrophages per unit area was measured in at least 10 random lesions from the carotid artery. For immunofluorescence, sections were deparaffinized and stained with antibodies specific for CD68, Mac-3 or CD146 followed by fluorescence-labeled secondary antibodies. The nuclei were counterstained with 4′,6-diamidino-2-phenylindole (DAPI). The sections were visualized using a confocal laser scanning microscope (Olympus, Tokyo, Japan).

### Bone marrow transplantation and macrophage labeling and emigration assay

We used a macrophage tracking technique to specifically examine the role of CD146 in macrophage emigration. Briefly, circulating monocytes from ApoE^−/−^ mice that were fed with Western diet for 12 weeks were labeled *in vivo* by an i.v. tail injection of microspheres (YG-beads) diluted in sterile PBS (1:4) as described^18^. The labeling efficiency (the percentage of bead-positive blood monocytes) was measured by flow cytometry at the indicated days after the beads were injected (Supplementary information, Figure S6). Mice were divided into three groups. One group of mice was assessed for recruitment of labeled monocytes to atherosclerotic plaques after 10 days (baseline). Two other groups of mice were injected intraperitoneally on days 4, 7, 10 and 13 with either anti-CD146 mAb AA98 or the control mIgG (200 μg per mouse) and assessed on day 14 for the number of labeled macrophages that remained in the plaques. The number of beads was normalized to monocyte labeling efficiency. Over time, the bead content of a plaque decreases if the beads leave the plaque by hitching a ride in the same cells that brought them in (or if they were transferred to another monocyte-derived cell that then leaves the plaque). It is important that the tracer beads be nonbiodegradable, allowing for uncoupling of bead fate from macrophage death and clearance. Consequently, a decrease in bead number would effectively reflect the emigration of monocyte-derived cells from the plaque^4,18,67^.

Bone marrow cells from CD146^WT^ or CD146^M-KO^ mice were prepared for a transplant. Male ApoE^−/−^ mice (fed the Western diet for 12 weeks) were irradiated with 9.8 Gy and then injected intravenously with the prepared bone marrow cells (2 × 10^6^ per mouse). After feeding on the normal diet for 2 months, the transplant-recipient mice were switched to the Western diet and stayed on it for 12 weeks. After that, the mice were used in the atherosclerosis analysis or in the macrophage tracking experiment as described above.

### Statistical analysis

All experiments were performed independently at least for three times. The results are shown as the mean ± SEM. One- or two-way ANOVA test was used to compare differences between groups in the various experiments. Differences of a *P*-value < 0.05 were considered as statistically significant.

## Author Contributions

Yongting Luo, Hongxia Duan and Yining Qian designed and performed experiments, analyzed data and wrote the manuscript; Liqun Feng and Zhenzhen Wu performed experiments and analyzed data; Jing Feng, Dongling Yang and Zhihai Qin analyzed data; and Xiyun Yan initiated the study, designed experiments, reviewed the data and wrote the manuscript.

## Competing Financial Interests

The authors declare no competing financial interests.

## Supplementary Information

Supplementary information, Figure S1 **(A)** The CD146 promoter sequence with NF-κB binding sites boxed in red.

Supplementary information, Figure S2 Generation of macrophage-specific CD146 knockout mice.

Supplementary information, Figure S3 Western blot analysis of CD146 expression in oxLDL-stimulated (50 μg/ml) BMDMs isolated from CD146^WT^ or CD146^M-KO^ mice.

Supplementary information, Figure S4 NF-κB activation in response to oxLDL is CD36-dependent.

Supplementary information, Figure S5 CD146 is not required for LPS- or TNFα-induced NF-κB activation.

Supplementary information, Figure S6 CD146 is not required for binding of oxLDL to macrophages.

Supplementary information, Figure S7 CD36 but not CD146 binds to oxLDL.

Supplementary information, Figure S8 Immunoblot analysis of gradient fractions with markers (Rab5, Rab7, and LAMP1) specific to endosomes.

Supplementary information, Figure S9 The internalization of CD36 and CD146 was detected by fluorescence microscopy.

Supplementary information, Figure S10 Quantitative real-time RT-PCR analysis of mRNA levels of the macrophage migratory factors *Cd36, Netrin-1, Sema 3E* and *Ccr7* in BMDMs that were incubated with oxLDL (50 μg/ml) for 24 h in the presence or absence of NF-κB inhibitor BAY11-7082 (20 μM).

Supplementary information, Figure S11 (**A**) Quantification of the number of bead-labeled macrophages in atherosclerotic plaques of ApoE^−/−^ mice in a monocyte bead-tracking model.

Supplementary information, Figure S12 Metabolic parameters (**A**) and body weight (**B**) of CD146^WT^→ApoE^−/−^ and CD146^M-KO^→ApoE^−/−^ chimeric mice (n = 10) that fed a Western diet for 12 weeks.

Supplementary information, Figure S13 The anti-CD146 monoclonal antibody AA98 recognizes murine CD146.

Supplementary information, Figure S14 Metabolic parameters and body weight of ApoE^−/−^ mice that were preventively (n = 8) (**A, B**) or therapeutically (n = 5) (**C, D**) injected with mIgG or anti-CD146 AA98.

Supplementary information, Figure S15 Quantitative real-time RT-PCR analysis of mRNA levels of *Cd36* in BMDMs (isolated from CD146^M-KO^ mice) that were treated with oxLDL (50 μg/ml) for 24 h in the presence or absence of the PPARγ antagonist T0070907 (1 μM).

Supplementary information, Figure S16 Immunofluorescent staining of atherosclerotic lesions isolated from CD146^WT^→ApoE^−/−^ or CD146^M-KO^→ApoE^−/−^ mice or mice preventively or therapeutically injected with the anti-CD146 antibody.

Supplementary information, Table S1 Real-time PCR primers used in this study
