# Supplementary material for: Macrophagic CD146 promotes foam cell formation and retention during atherosclerosis
Source: Cell Res. 2017 Jan 13;27(3):352–72. doi: 10.1038/cr.2017.8 (PMC5339843; doi:10.1038/cr.2017.8)
Supplement: Supplementary information, Figure S1 — (A) The CD146 promoter sequence with NF-κB binding sites boxed in red. [file cr20178x1.pdf]

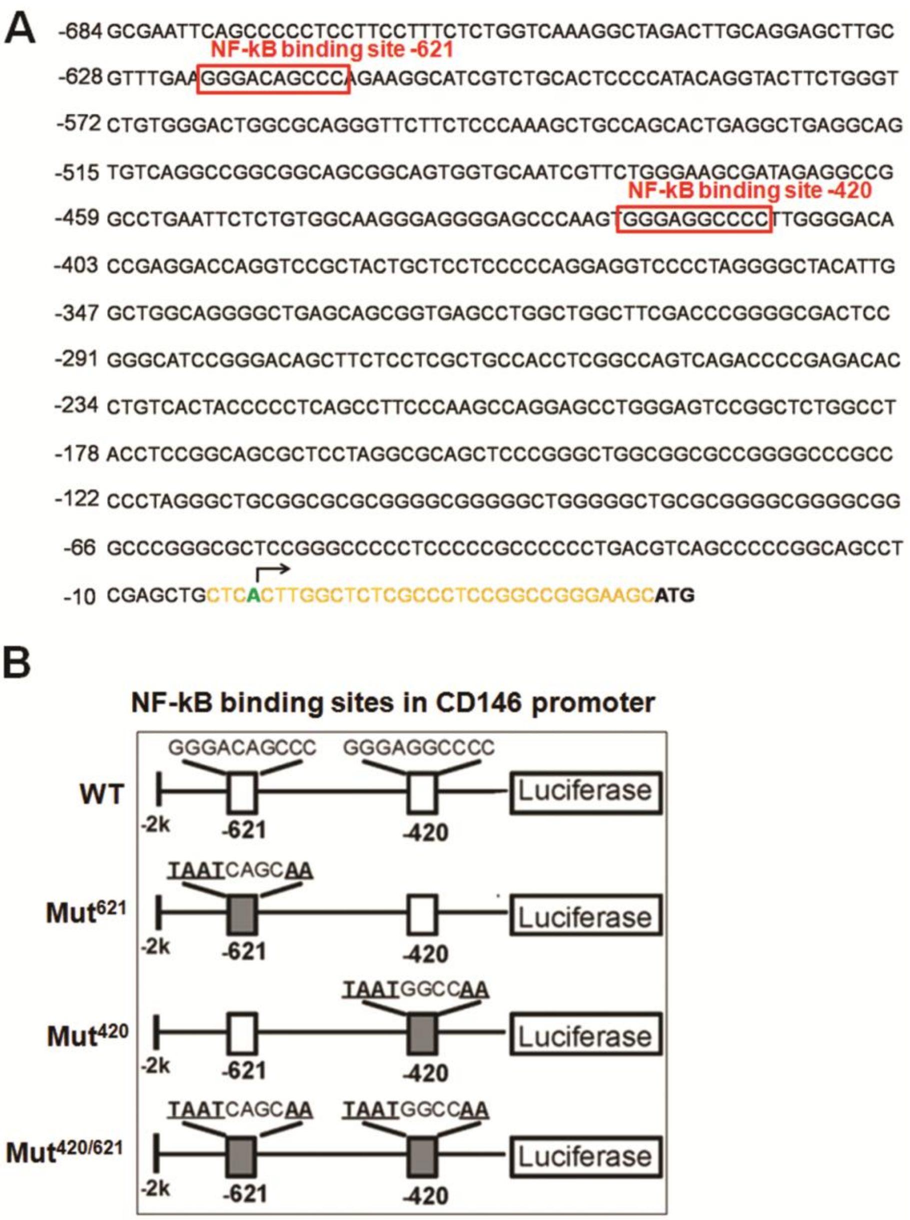


**Supplementary information, Figure S1** (**A**) The CD146 promoter sequence with NF-κB binding sites boxed in red. (**B**) Schematic overview of mutant constructs of NF-κB binding sites is shown.
