# Supplementary material for: Macrophagic CD146 promotes foam cell formation and retention during atherosclerosis
Source: Cell Res. 2017 Jan 13;27(3):352–72. doi: 10.1038/cr.2017.8 (PMC5339843; doi:10.1038/cr.2017.8)
Supplement: Supplementary information, Figure S2 — Generation of macrophage-specific CD146 knockout mice. [file cr20178x2.pdf]

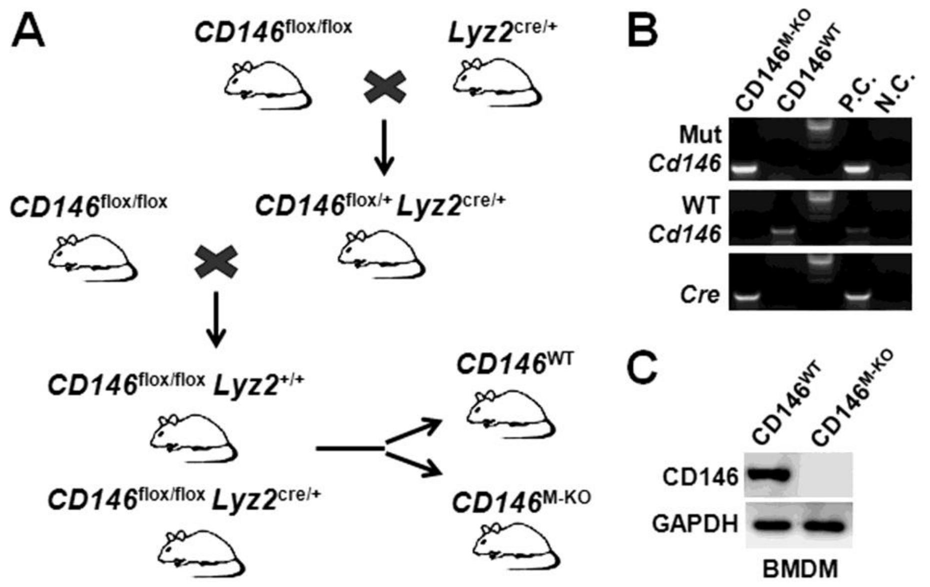

Supplementary information, Figure S2 Generation of macrophage-specific CD146 knockout mice. (**A**) Mating scheme to generate macrophage-specific CD146 knockout mice (Lyz2$^{cre/+}$CD146$^{flox/flox}$, namely CD146$^{M\text{-}KO}$) and control WT littermates (Lyz2$^{+/+}$CD146$^{flox/flox}$, namely CD146^{WT}). (**B**) Genotyping of CD146$^{M\text{-}KO}$ and CD146^{WT}mice by PCR analysis of genomic DNA. A 595-bp fragment from wild-type *Cre* gene, a 550-bp fragment from wild-type *Cd146* gene (WT *Cd146*) and a 502-bp fragment from floxed *Cd146* gene (Mut *Cd146*) were PCR-amplified with specific primers. Genomic DNA from Lyz2$^{cre/+}$ mice was used as positive control (P.C.) for *Cre* analysis; Genomic DNA from CD146$^{floxed/floxed}$ mice were used as P.C. for Mu *Cd146* analysis; genomic DNA from C57BL/6 mice were used as P.C. for WT *Cd146* analysis. H_2O was used as negative control (N.C.) for all three PCR analyses. (**C**) Characterization of BMDMs isolated from CD146^{WT} or CD146$^{M\text{-}KO}$ mice by western blot using specific antibodies as indicated.
