# Supplementary material for: Macrophagic CD146 promotes foam cell formation and retention during atherosclerosis
Source: Cell Res. 2017 Jan 13;27(3):352–72. doi: 10.1038/cr.2017.8 (PMC5339843; doi:10.1038/cr.2017.8)
Supplement: Supplementary information, Figure S3 — Western blot analysis of CD146 expression in oxLDL-stimulated (50 μg/ml) BMDMs isolated from CD146WT or CD146M-KO mice. [file cr20178x3.pdf]

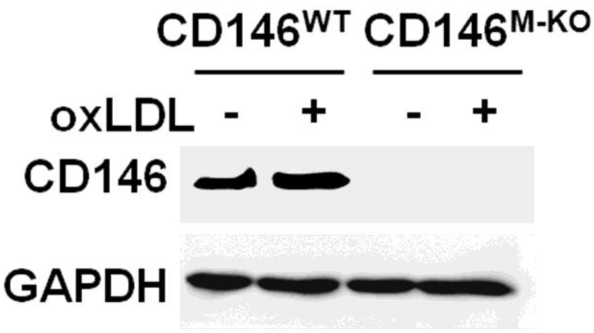

Supplementary information, Figure S3 Western blot analysis of CD146 expression in oxLDL-stimulated (50 μg/ml) BMDMs isolated from $CD146^{WT}$ or $CD146^{M\text{-}KO}$ mice. GAPDH was used as a loading control. The data represent three independent experiments.
