# Supplementary material for: Macrophagic CD146 promotes foam cell formation and retention during atherosclerosis
Source: Cell Res. 2017 Jan 13;27(3):352–72. doi: 10.1038/cr.2017.8 (PMC5339843; doi:10.1038/cr.2017.8)
Supplement: Supplementary information, Figure S4 — NF-κB activation in response to oxLDL is CD36-dependent. [file cr20178x4.pdf]

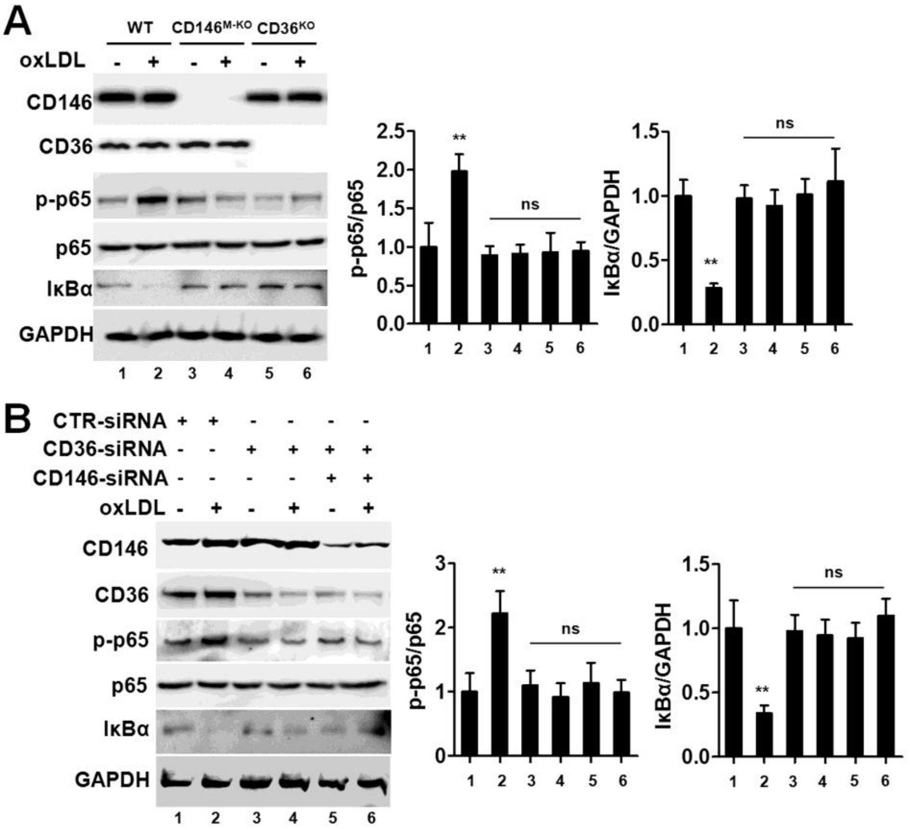

Supplementary information, Figure S4 NF-κB activation in response to oxLDL is CD36-dependent. (**A, B**) Western blot analysis of IκBα, phosphorylated (p-) and total NF-κB p65 in oxLDL- (50 ng/ml) stimulated BMDMs isolated from WT (wild type), CD146$^{M\text{-}KO}$ and CD36^{KO} mice (**A**) or WT BMDMs with or without pretreatment with anti-CD146 AA98 (50 μg/ml) (**B**). GAPDH was used as a loading control. Right panel: the quantification of relative expression of p-p65 and IκBα. Two-way ANOVA test. $^{**}P < 0.01$. The data represent three independent experiments.
