# Supplementary material for: Macrophagic CD146 promotes foam cell formation and retention during atherosclerosis
Source: Cell Res. 2017 Jan 13;27(3):352–72. doi: 10.1038/cr.2017.8 (PMC5339843; doi:10.1038/cr.2017.8)
Supplement: Supplementary information, Figure S5 — CD146 is not required for LPS- or TNFα-induced NF-κB activation. [file cr20178x5.pdf]

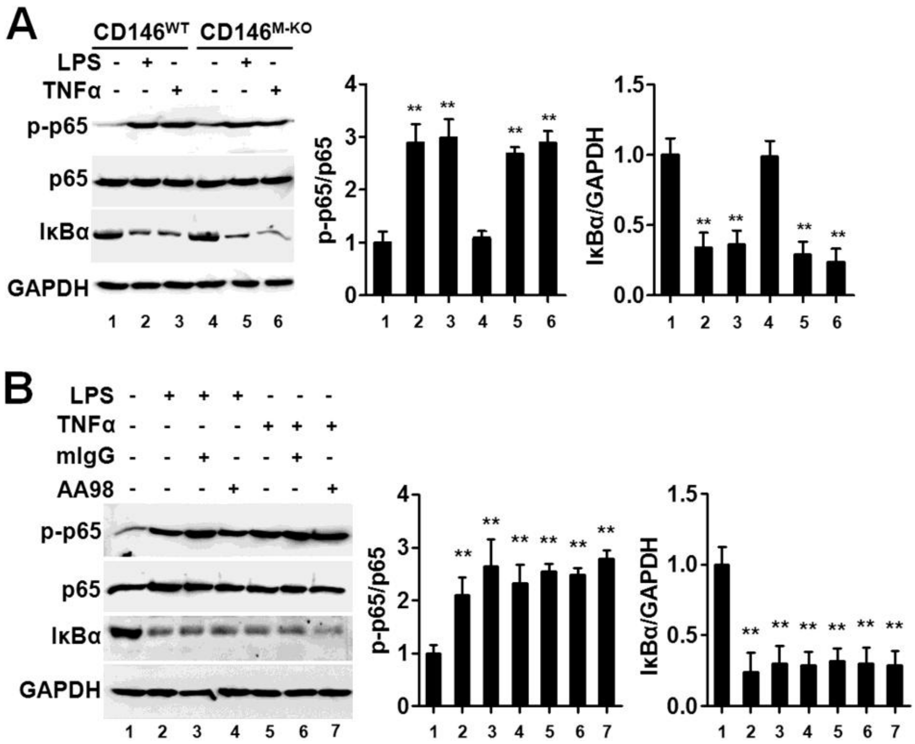

Supplementary information, Figure S5 CD146 is not required for LPS- or TNFα-induced NF-κB activation. (**A, B**) Western blot analysis of IκBα, phosphorylated (p-) and total NF-κB p65 in LPS- (10 μg/ml) or TNFα- (50 ng/ml) stimulated BMDMs isolated from CD146^{WT} or CD146$^{M\text{-}KO}$ mice (**A**) or BMDMs with or without pretreatment with anti-CD146 AA98 (50 μg/ml) (**B**). GAPDH was used as a loading control. Right panel: the quantification of relative expression of p-p65 and IκBα. Two-way ANOVA test. ***P* < 0.01. The data represent three independent experiments.
