# Supplementary material for: Macrophagic CD146 promotes foam cell formation and retention during atherosclerosis
Source: Cell Res. 2017 Jan 13;27(3):352–72. doi: 10.1038/cr.2017.8 (PMC5339843; doi:10.1038/cr.2017.8)
Supplement: Supplementary information, Figure S6 — CD146 is not required for binding of oxLDL to macrophages. [file cr20178x6.pdf]

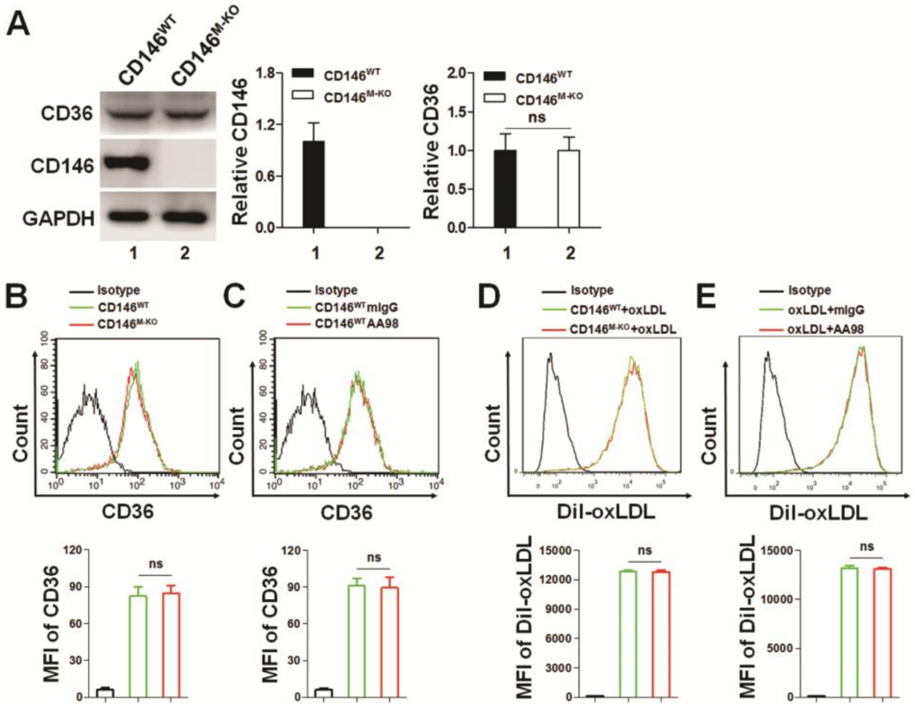

Supplementary information, Figure S6 CD146 is not required for binding of oxLDL to macrophages. (**A**) Western blot analysis of CD146 and CD36 expression in BMDMs isolated from CD146^{WT} or CD146$^{M\text{-}KO}$ mice. GAPDH served as a loading control. Right panel: quantification of relative expression of CD146 and CD36. (**B**) The BMDMs were incubated with oxLDL at 4 °C for 15 min to prevent endocytosis. Flow cytometric analysis of CD36 expression in BMDMs isolated from CD146^{WT} or CD146$^{M\text{-}KO}$ mice. (**C**) Flow cytometric analysis of CD36 expression in BMDMs that were treated with mIgG or anti-CD146 AA98 (50 μg/ml). Bottom panel: quantification of the MFI of CD36 in each group (n = 5). (**D**) The BMDMs were incubated with Dil-oxLDL at 4 °C for 15 min to prevent endocytosis. Flow cytometric analysis of the binding of Dil-oxLDL to BMDMs isolated from CD146^{WT} or CD146$^{M\text{-}KO}$ mice. (**E**) Flow cytometric analysis of the binding of Dil-oxLDL to BMDMs that were treated with mIgG or anti-CD146 AA98 (50 μg/ml). Bottom panel: quantification of the MFI of Dil-oxLDL in each group (n = 5). The data represent three independent experiments.
