# Supplementary material for: Macrophagic CD146 promotes foam cell formation and retention during atherosclerosis
Source: Cell Res. 2017 Jan 13;27(3):352–72. doi: 10.1038/cr.2017.8 (PMC5339843; doi:10.1038/cr.2017.8)
Supplement: Supplementary information, Figure S7 — CD36 but not CD146 binds to oxLDL. [file cr20178x7.pdf]

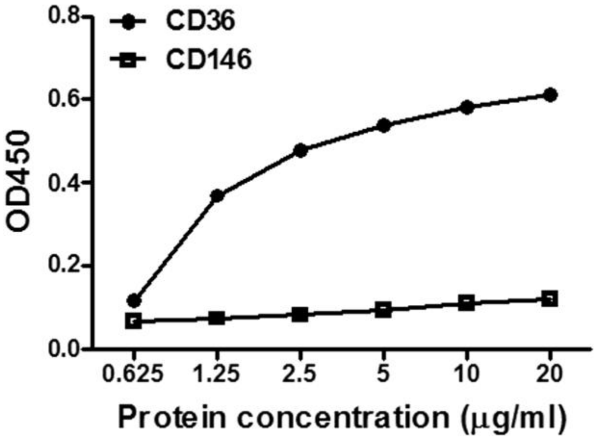

Supplementary information, Figure S7 CD36 but not CD146 binds to oxLDL. Different concentrations of His-tagged CD36 or CD146 were added to wells coated with oxLDL (5 μg/ml), and enzyme-linked immunosorbent assay was performed. The data represent three independent experiments.
