# Supplementary material for: Macrophagic CD146 promotes foam cell formation and retention during atherosclerosis
Source: Cell Res. 2017 Jan 13;27(3):352–72. doi: 10.1038/cr.2017.8 (PMC5339843; doi:10.1038/cr.2017.8)
Supplement: Supplementary information, Figure S8 — Immunoblot analysis of gradient fractions with markers (Rab5, Rab7, and LAMP1) specific to endosomes. [file cr20178x8.pdf]

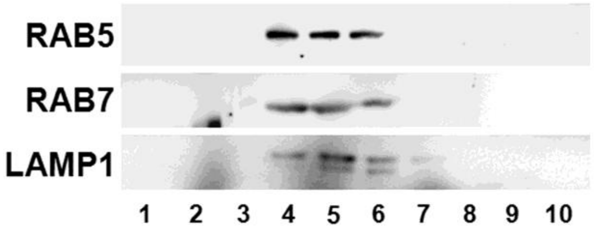

Supplementary information, Figure S8 Immunoblot analysis of gradient fractions with markers (Rab5, Rab7, and LAMP1) specific to endosomes. The endosomal fractions were isolated by differential centrifugation in a discontinuous sucrose gradient (consisting of three steps: 8%, 35%, and 42% sucrose). The data represent three independent experiments.
