# Supplementary material for: Macrophagic CD146 promotes foam cell formation and retention during atherosclerosis
Source: Cell Res. 2017 Jan 13;27(3):352–72. doi: 10.1038/cr.2017.8 (PMC5339843; doi:10.1038/cr.2017.8)
Supplement: Supplementary information, Figure S9 — The internalization of CD36 and CD146 was detected by fluorescence microscopy. [file cr20178x9.pdf]

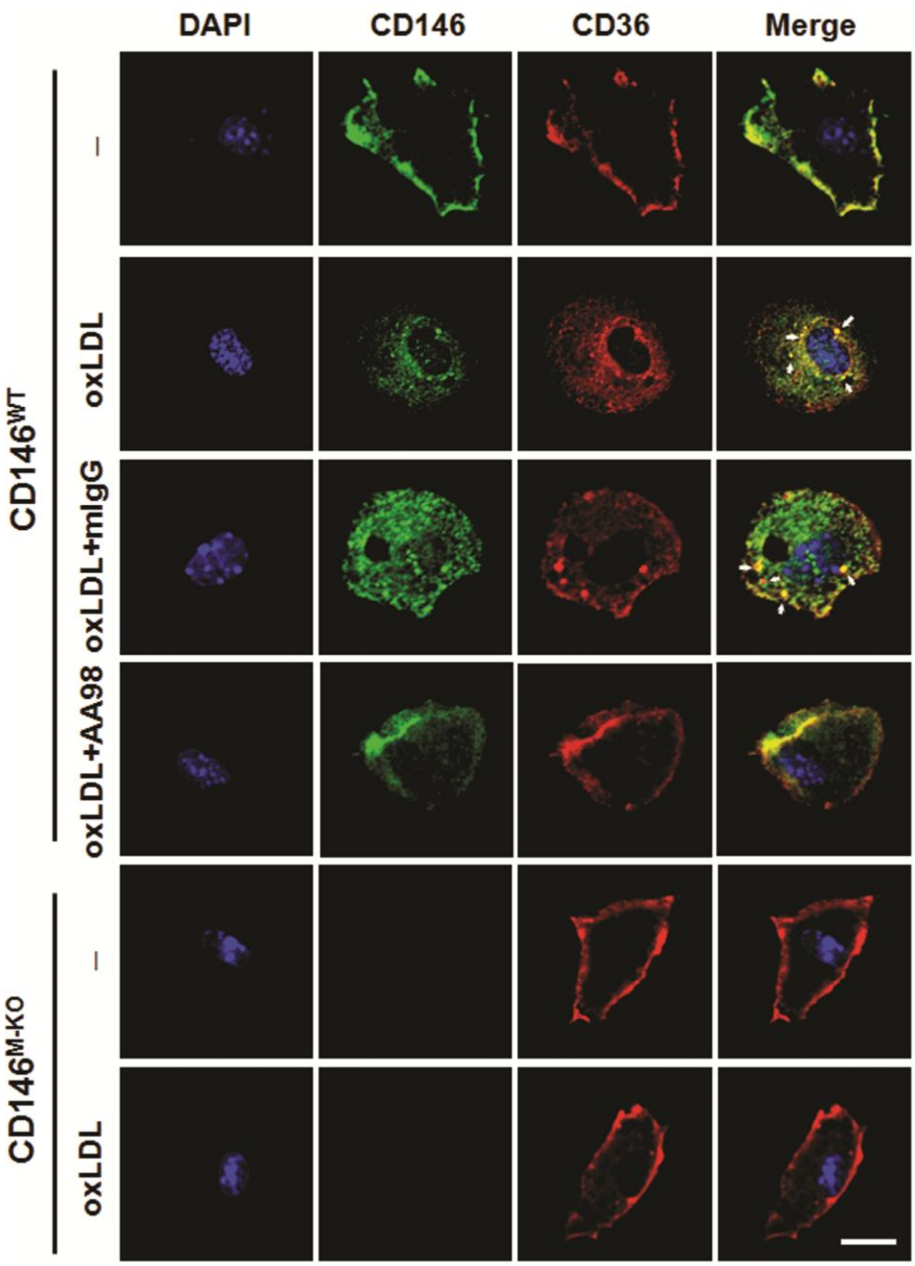

Supplementary information, Figure S9 The internalization of CD36 and CD146 was detected by fluorescence microscopy. BMEMs were treated or not treated with oxLDL for 15 min and stained for CD146 (green) and CD36 (red). The nuclei were stained with DAPI (blue). The colocalization of CD36 and CD146 after stimulation with oxLDL was examined in endosome like structures (white arrowheads). The scale bar is 20 μm. The data represent three independent experiments.
