# Supplementary material for: Macrophagic CD146 promotes foam cell formation and retention during atherosclerosis
Source: Cell Res. 2017 Jan 13;27(3):352–72. doi: 10.1038/cr.2017.8 (PMC5339843; doi:10.1038/cr.2017.8)
Supplement: Supplementary information, Figure S10 — Quantitative real-time RT-PCR analysis of mRNA levels of the macrophage migratory factors Cd36, Netrin-1, Sema 3E and Ccr7 in BMDMs that were incubated with oxLDL (50 μg/ml) for 24 h in the presence or absence of NF-κB inhibitor BAY11-7082 (20 μM). [file cr20178x10.pdf]

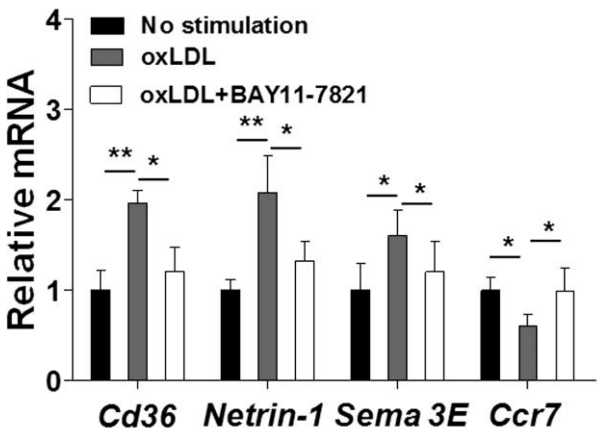

Supplementary information, Figure S10 Quantitative real-time RT-PCR analysis of mRNA levels of the macrophage migratory factors *Cd36, Netrin-1, Sema 3E* and *Ccr7* in BMDMs that were incubated with oxLDL (50 μg/ml) for 24 h in the presence or absence of NF-κB inhibitor BAY11-7082 (20 μM). **P* < 0.05, ***P* < 0.01. The data represent three independent experiments.
