# Supplementary material for: Macrophagic CD146 promotes foam cell formation and retention during atherosclerosis
Source: Cell Res. 2017 Jan 13;27(3):352–72. doi: 10.1038/cr.2017.8 (PMC5339843; doi:10.1038/cr.2017.8)
Supplement: Supplementary information, Figure S11 — (A) Quantification of the number of bead-labeled macrophages in atherosclerotic plaques of ApoE−/− mice in a monocyte bead-tracking model. [file cr20178x11.pdf]

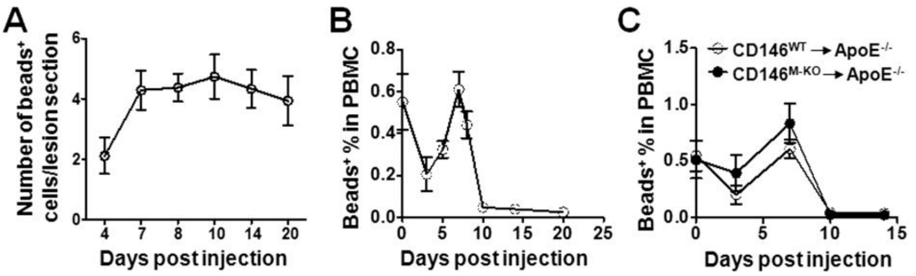

Supplementary information, Figure S11 (**A**) Quantification of the number of bead-labeled macrophages in atherosclerotic plaques of ApoE$^{-/-}$ mice in a monocyte bead-tracking model. The data are presented as bead-labeled macrophages per plaque on the indicated days after monocyte labeling. (**B**) Quantification of the bead^{+} cells among peripheral blood mononuclear cells (PBMCs) of ApoE$^{-/-}$ mice on the indicated days after bead transfer in the monocyte bead-tracking model. (**C**) Quantification of the bead^{+} cells among PBMCs of CD146^{WT}→ApoE$^{-/-}$ or CD146$^{M\text{-}KO}$→ApoE$^{-/-}$ chimeric mice on the indicated days after bead transfer. Data are representative of one experiment each with five mice for each indicated day (**A, B**) or six mice per genotype (**C**).
