# Supplementary material for: Macrophagic CD146 promotes foam cell formation and retention during atherosclerosis
Source: Cell Res. 2017 Jan 13;27(3):352–72. doi: 10.1038/cr.2017.8 (PMC5339843; doi:10.1038/cr.2017.8)
Supplement: Supplementary information, Figure S12 — Metabolic parameters (A) and body weight (B) of CD146WT→ApoE−/− and CD146M-KO→ApoE−/− chimeric mice (n = 10) that fed a Western diet for 12 weeks. [file cr20178x12.pdf]

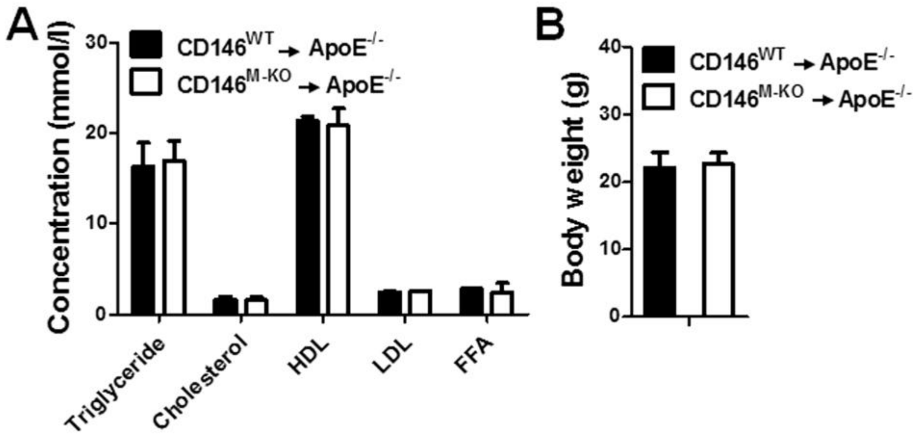

Supplementary information, Figure S12 Metabolic parameters (**A**) and body weight (**B**) of $CD146^{WT}$→$ApoE^{-/-}$ and $CD146^{M\text{-}KO}$→$ApoE^{-/-}$ chimeric mice (n = 10) that fed a Western diet for 12 weeks.
