# Supplementary material for: Macrophagic CD146 promotes foam cell formation and retention during atherosclerosis
Source: Cell Res. 2017 Jan 13;27(3):352–72. doi: 10.1038/cr.2017.8 (PMC5339843; doi:10.1038/cr.2017.8)
Supplement: Supplementary information, Figure S13 — The anti-CD146 monoclonal antibody AA98 recognizes murine CD146. [file cr20178x13.pdf]

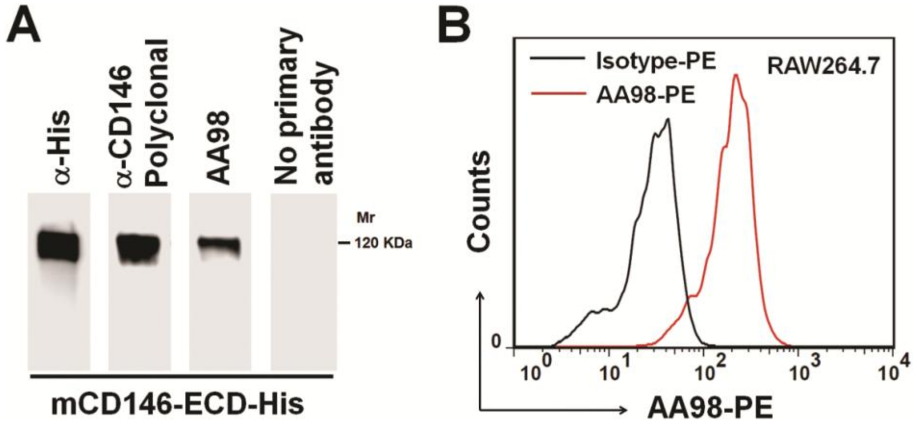

Supplementary information, Figure S13 The anti-CD146 monoclonal antibody AA98 recognizes murine CD146. (**A**) His-tagged murine CD146 protein was detected with antibodies of anti-His-tag, anti-CD146 polyclonal antibody, and AA98. (**B**) The CD146 expression in RAW264.7 cells was detected with AA98-PE. The data represent three independent experiments.
