# Supplementary material for: Macrophagic CD146 promotes foam cell formation and retention during atherosclerosis
Source: Cell Res. 2017 Jan 13;27(3):352–72. doi: 10.1038/cr.2017.8 (PMC5339843; doi:10.1038/cr.2017.8)
Supplement: Supplementary information, Figure S14 — Metabolic parameters and body weight of ApoE−/− mice that were preventively (n = 8) (A, B) or therapeutically (n = 5) (C, D) injected with mIgG or anti-CD146 AA98. [file cr20178x14.pdf]

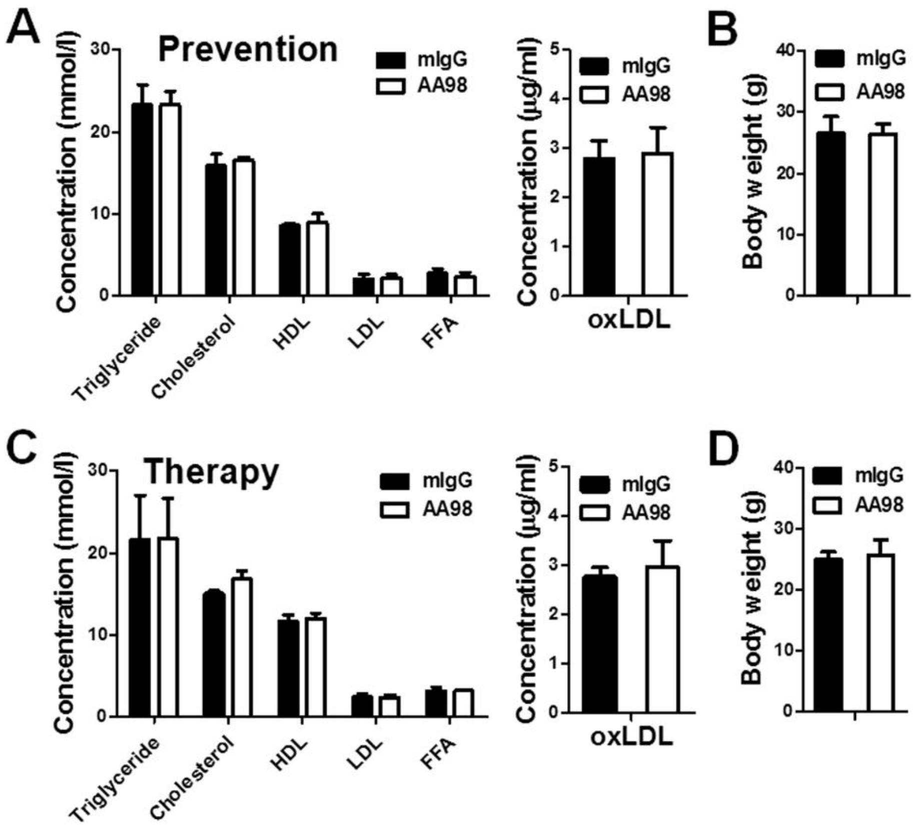

Supplementary information, Figure S14 Metabolic parameters and body weight of ApoE$^{-/-}$ mice that were preventively (n = 8) (**A, B**) or therapeutically (n = 5) (**C, D**) injected with mIgG or anti-CD146 AA98.
