# Supplementary material for: Macrophagic CD146 promotes foam cell formation and retention during atherosclerosis
Source: Cell Res. 2017 Jan 13;27(3):352–72. doi: 10.1038/cr.2017.8 (PMC5339843; doi:10.1038/cr.2017.8)
Supplement: Supplementary information, Figure S15 — Quantitative real-time RT-PCR analysis of mRNA levels of Cd36 in BMDMs (isolated from CD146M-KO mice) that were treated with oxLDL (50 μg/ml) for 24 h in the presence or absence of the PPARγ antagonist T0070907 (1 μM). [file cr20178x15.pdf]

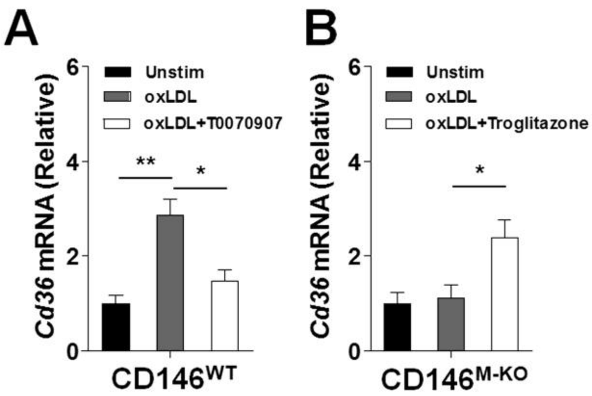

Supplementary information, Figure S15 (**A**) Quantitative real-time RT-PCR analysis of mRNA levels of *Cd36* in BMDMs (isolated from CD146$^{M\text{-}KO}$ mice) that were treated with oxLDL (50 μg/ml) for 24 h in the presence or absence of the PPARγ antagonist T0070907 (1 μM). (**B**) Quantitative real-time RT-PCR analysis of mRNA levels of *Cd36* in BMDMs (isolated from CD146$^{M\text{-}KO}$ mice) that were treated with oxLDL (50 μg/ml) for 24 h in the presence or absence of the PPARγ agonist troglitazone (40 μM). *$P < 0.05$, **$P < 0.01$. The data represent three independent experiments.
