# Supplementary material for: Macrophagic CD146 promotes foam cell formation and retention during atherosclerosis
Source: Cell Res. 2017 Jan 13;27(3):352–72. doi: 10.1038/cr.2017.8 (PMC5339843; doi:10.1038/cr.2017.8)
Supplement: Supplementary information, Figure S16 — Immunofluorescent staining of atherosclerotic lesions isolated from CD146WT→ApoE−/− or CD146M-KO→ApoE−/− mice or mice preventively or therapeutically injected with the anti-CD146 antibody. [file cr20178x16.pdf]

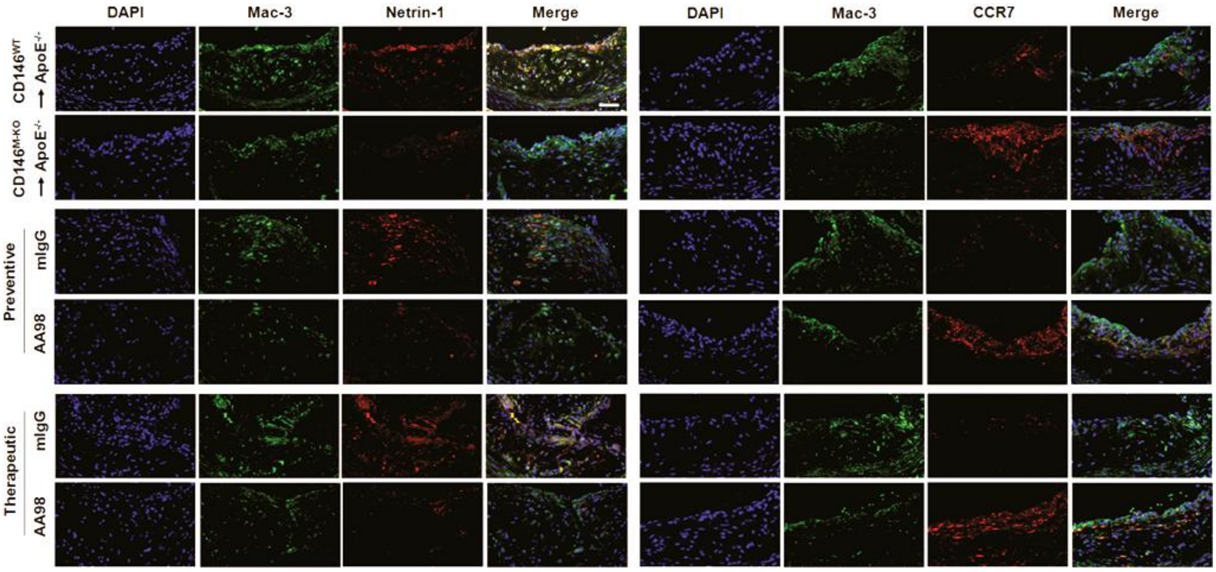

Supplementary information, Figure S16 Immunofluorescent staining of atherosclerotic lesions isolated from $CD146^{WT}$→$ApoE^{-/-}$ or $CD146^{M\text{-}KO}$→$ApoE^{-/-}$ mice or mice preventively or therapeutically injected with the anti-CD146 antibody. The lesions were costained for Mac-3 (green) and netrin-1 or CCR7 (red). The nuclei were stained with DAPI (blue). The scale bar is 50 μm. The data represent three independent experiments.
