# Supplementary material for: Macrophagic CD146 promotes foam cell formation and retention during atherosclerosis
Source: Cell Res. 2017 Jan 13;27(3):352–72. doi: 10.1038/cr.2017.8 (PMC5339843; doi:10.1038/cr.2017.8)
Supplement: Supplementary information, Table S1 — Real-time PCR primers used in this study [file cr20178x17.pdf]

Supplementary information, Table S1 Real-time PCR primers used in this study

Target genes	sequence (5'-3')
**GAPDH**	Forward：GGGAAATGAGAGAGGCCCAG
	Reverse：GGCACTGCACAAGAAGATGC
**CD146**	Forward：CGGGTGTGCCAGGAGAG
	Reverse：GGCGGTGCTCATATTCACCA
**MCP-1**	Forward：GACCCCAAGAAGGAATGGGT
	Reverse：ACAGAAGTGCTTGAGGTGGTT
**MMP-9**	Forward：CCAGCCGACTTTTGTGGTCT
	Reverse：TGGCCTTTAGTGTCTGGCTG
**TNFα**	Forward：GTAGCCCACGTCGTAGCAAA
	Reverse：TAGCAAATCGGCTGACGGTG
**IFNγ**	Forward：ATTGCGGGGTTGTATCTGGG
	Reverse：ACATTCGAGTGCTGTCTGGC
**IL1-β**	Forward：AAGCACCAGCACATTGCTTT
	Reverse：TGTTGCCCTCAGCAGTAAGG
**Netrin-1**	Forward：CGTGGTGAAGAGCGCGT
	Reverse：GTCACCTCAAACTTCTTGCCG
**CD36**	Forward：CTCCTAGTAGGCGTGGGTCT
	Reverse：TGGCTTCAGGGAGACTGTTG
**CCR7**	Forward：GTGGTGGCTCTCCTTGTCATT
	Reverse：GTACGTCAGTATCACCAGCCC
**Sema 3E**	Forward：TATGGCAGTGCTTGTGCTGA
	Reverse：TCTTCAGTCCTGTCCAACGC
